# Triple‐Functional Cu_x_Au_61‐x_ Nanoclusters with NIR‐II Photoluminescence, Photothermal and Photodynamic Properties and Their Bio‐Application

**DOI:** 10.1002/advs.202509283

**Published:** 2025-07-18

**Authors:** Tingting Xu, Jie Kong, Yu Chen, Wenxue Cui, Yucheng Fang, Wei Zhang, Mohan Wang, Meng Zhou, Yingwei Li, Rongchao Jin, Yongbo Song

**Affiliations:** ^1^ School of Biomedical Engineering, Research and Engineering Center of Biomedical Materials Anhui Medical University Hefei Anhui 230032 P. R. China; ^2^ Hefei National Research Center for Physical Sciences at the Microscale University of Science and Technology of China Hefei Anhui 230026 P. R. China; ^3^ National Clinical Research Centre for Oral Diseases, Shanghai Key Laboratory of Stomatology and Shanghai Research Institute of Stomatology, Department of Oral Surgery Shanghai Ninth People's Hospital College of Stomatology Shanghai Jiao Tong University School of Medicine Shanghai 200011 P. R. China; ^4^ Department of Chemistry Carnegie Mellon University Pittsburgh PA 15213 USA

**Keywords:** atomically precise nanoclusters, NIR‐II photoluminescence, photodynamic, photothermal, structure

## Abstract

Atomically precise gold nanoclusters (NCs) hold great potential in optical and bio‐related applications due to their well‐defined atomic structures, near‐infrared‐II (NIR‐II) photoluminescence, and excellent biocompatibility. However, many applications require multi‐functionality in one entity, which poses significant challenges in the design of materials. Herein, a novel Cu_x_Au_61‐x_ NC is designed, exhibiting triple functionality, including NIR‐II luminescence, strong photothermal effect, and high catalytic activity. Crystallographic analysis reveals a penta‐icosahedral, oblate structure, with the Cu atoms being exclusively located in the core region, including one additional Cu atom at the center of the Cu_x_Au_61‐x_ structure. By comparing with the related, undoped Au_60_ NC —which is non‐emissive and photocatalytically inactive, deep insights into the enhancement mechanisms are obtained. Furthermore, a multifunctional Cu_x_Au_61‐x_@SiO_2_‐FA (folic acid) nanoplatform is constructed for potential bio‐applications.

## Graphical abstract

A new Cu_x_Au_61‐x_ nanocluster was synthesized by doping Cu atoms into the Au_60_ nanocluster, and “one stone two birds” was achieved, in which both the NIR‐II emission and ROS generation of the Cu_x_Au_61‐x_ nanocluster were significantly enhanced. Considering its photothermal effect, a Cu_x_Au_61‐x_@SiO_2_‐FA nanoplatform was constructed for NIR‐II luminescence imaging‐guided tumor therapy.

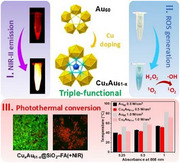

## 1 Introduction

Atomically precise metal nanoclusters (NCs) have emerged as a novel class of functional nanomaterials. Their ultrasmall sizes impart molecule‐like discrete electronic energy states, offering significant potential in various photon‐induced applications, including photocatalysis,^[^ ^1^ ^]^ biosensing,^[^ ^2^, ^3^ ^]^ bioimaging,^[^ ^4^, ^5^, ^6^ ^]^ and chiroptical emission.^[^ ^7^, ^8^ ^]^ Among these, the excellent optical properties of metal NCs have shown considerable promise in biomedicine.^[^ ^9^, ^10^, ^11^, ^12^ ^]^ With advances in understanding structure‐activity relationships and synthetic strategies, an increasing number of NCs with second near‐infrared (NIR‐II, 1000–1700 nm) luminescence have been developed.^[^ ^13^, ^14^, ^15^, ^16^ ^]^ Notably, NIR‐II emitters offer a large Stokes shift, good photostability, and multi‐wavelength excitation capabilities, all of which are crucial for improving the signal‐to‐background ratio (SBR) and imaging resolution,^[^ ^17^, ^18^ ^]^ making them particularly attractive for NIR‐II bioimaging.^[^ ^19^, ^20^ ^]^ In addition, some molecule‐like metal NCs exhibit strong photothermal conversion efficiency and can be applied to photothermal therapy (PTT) due to their broad absorption range spanning from the UV to NIR‐II region, providing greater flexibility in irradiation wavelength selection.^[^ ^21^, ^22^, ^23^ ^]^ Furthermore, their high specific surface area and abundant free electrons confer enzyme‐like catalytic activity, enabling applications in chemodynamic therapy (CDT) and photodynamic therapy (PDT).^[^ ^24^, ^25^ ^]^ Overall, atomically precise metal NCs present significant potential for precise tumor therapy guided by NIR‐II imaging.^[^ ^26^, ^27^, ^28^, ^29^ ^]^ However, a major challenge remains in the rational design of metal NCs that integrate all these functionalities to achieve comprehensive biomedical applications.

Malignant tumors represent one of the most formidable global health challenges, and significant efforts have been dedicated to developing innovative treatment strategies that specifically address the complex and dynamic nature of tumor pathogenesis.^[^ ^30^, ^31^, ^32^ ^]^ Luminescence imaging‐guided phototherapy is a non‐invasive and highly efficient antitumor approach.^[^ ^33^, ^34^ ^]^ In this strategy, laser irradiation not only allows for precise localization of drug delivery to the tumor site and real‐time visualization of tumor shape and size via near‐infrared luminescence imaging but also facilitates antitumor treatment through PTT or PDT.^[^ ^35^, ^36^, ^37^ ^]^ Notably, NIR‐II photoluminescence (PL) imaging exhibits superior spatial resolution and deeper tissue penetration compared to first near‐infrared region (NIR‐I, 700−900 nm) and visible light imaging. This advantage arises from reduced tissue scattering and absorption, resulting in minimal attenuation—an essential feature for clinical in vivo applications.^[^ ^38^, ^39^, ^40^ ^]^ However, most of the developed probes for NIR‐II PL imaging‐guided phototherapy suffer from small Stokes shifts or poor photostability.^[^ ^41^ ^]^ In this context, metal NCs offer new opportunities as the next‐generation probe, potentially overcoming these limitations and advancing the field of precision phototherapy.

In this study, we report a multifunctional [Cu_x_Au_61‐x_Se_2_(Ph_3_P)_10_(SePh)_15_]^2+^ alloy NC (abbrev. Cu_x_Au_61‐x_) with x = 1–11. Our strategy of Cu doping into the parent [Au_60_Se_2_(Ph_3_P)_10_(SePh)_15_]^+^ NC (abbrev. Au_60_) significantly enhances the NIR‐II emission and singlet oxygen generation capability of the NC. Single‐crystal X‐ray diffraction reveals that both Cu_x_Au_61‐x_ and Au_60_ NCs possess a penta‐icosahedral structure, and the Cu atoms are exclusively located in the core of the alloy NC, which is against the common observation that atoms of a more active metal should go to the surface. With the significantly enhanced NIR‐II emission, the strong capability of photocatalytic generation of reactive oxygen species (ROS), and the impressive photothermal effect, this alloy NC acquires triple functionality, making it well‐suited for NIR‐II luminescence imaging‐guided tumor therapy. A multifunctional Cu_x_Au_61‐x_@SiO_2_‐FA nanoplatform is developed, demonstrating effective tumor inhibition and high biosafety both in vitro and in vivo.

## 2 Results and Discussion

### 2.1 Synthesis and Crystal structure of Cu_x_Au_61‐x_ NC

The Cu_x_Au_61‐x_ NC was synthesized using a metal‐exchange method.^[^ ^42^ ^]^ Briefly, Au_60_ NC was prepared according to our previous method.^[^ ^43^ ^]^ Subsequently, 20 mg of the NCs was dissolved in toluene (10 mL), followed by the addition of 10 mg of Ph_3_P‐Cu(I)‐Br complex. The reaction was allowed to proceed for ≈12 h under stirring (≈300 rpm) at room temperature. The product was washed thoroughly with hexane, extracted with toluene, and recrystallized in *n*‐hexane/toluene at 4 °C. Black needle‐like crystals were obtained after a week (yield: 63%).

**Figure 1:**
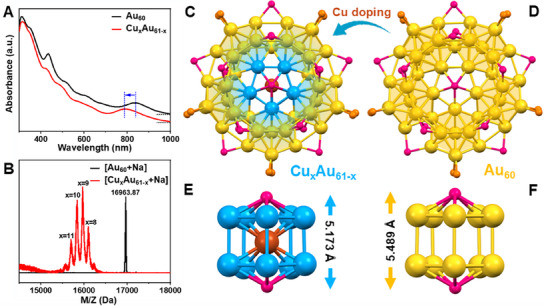
Characterization of Cu_x_Au_61‐x_ NC. A) UV–vis spectra of Au_60_ (black) and Cu_x_Au_61‐x_ (red) NCs in toluene. B) MALDI‐MS spectra of as‐synthesized Au_60_ (black) and Cu_x_Au_61‐x_ (red). Crystal structures of C) Cu_x_Au_61‐x_ and D) Au_60_, and E,F) the corresponding metal cores (side view) of the two NCs. Color code: yellow = Au; blue = Au/Cu (partial occupancies); brown = Cu; magenta = Se; orange = P, C, and H are omitted for clarity.

The Cu_x_Au_61‐x_ NC exhibits multiple absorption peaks at 420, 490, 575, and 790 nm in the UV–vis–NIR range (**Figure** **1**A, red line), with the longest‐wavelength absorption showing a significant blueshift compared to that of the parent Au_60_ template (Figure 1A, 790 vs 835 nm), a phenomenon also observed in other Cu‐doped Au NCs.^[^ ^44^ ^]^ Matrix‐assisted laser desorption ionization mass spectrometry (MALDI‐MS) spectrum of the Au_60_ NC shows a prominent peak for [Au_60_+Na] at m/z = 16963.87 (Figure 1B, black line). The alloy NCs exhibit a series of peaks ranging from 15500 to 16250 Da (Figure 1B, red line), with a mass interval (Δ*m*) of ≈134 Da, corresponding to the mass difference between Au and Cu, i.e., [Cu_x_Au_61‐x_+Na] with x = 8–11.

Single‐crystal X‐ray diffraction reveals that the Au_60_ template structure is retained in the 61‐atom alloy NC (Figure 1C,D), and the average Cu occupancy was determined to be x = 5.28 (Table S1, Supporting Information). Although the Cu_x_Au_61‐x_ NC possesses the same surface‐protecting layer as Au_60_ —, i.e., containing 10 Ph_3_P and 15 PhSe ligands as well as two Se atoms (Figure 1C,D)—one additional Cu (100% occupancy) is inserted into the central vacancy (Figure 1E,F). Furthermore, two Br^–^ counterions were observed along with each Cu_x_Au_61‐x_ NC (Figure S1, Supporting Information), hence, formulated as [Cu_x_Au_61‐x_Se_2_(PhSe)_15_(Ph_3_P)_10_]Br_2_. In both Cu_x_Au_61‐x_ and Au_60_ NCs, the oblate‐shaped NCs consist of five icosahedral units arranged in a cyclic manner by sharing one vertex metal atom (Figure 1C,D, highlighted in shading). The doped Cu atoms are located only within the inner ring of the penta‐icosahedral structure (the partial Cu occupancies are provided in Table S2, Supporting Information), surrounding the additional central Cu atom (Figure 1E), despite the previously observed general tendency of more electronegative atoms occupying the inner sites (*χ*_Cu_ = 1.90, *χ*_Au_ = 2.54).^[^ ^45^, ^46^ ^]^ The filling of the central vacancy in Au_60_ by an additional Cu is attributed to the much smaller atomic radius of Cu (1.28 vs 1.44 Å for Au), which is consistent with our previous observation in the Au_52_Cu_72_ NC, where two central Cu atoms facilitate the extension of a truncated Marks decahedron into a full decahedron.^[^ ^47^ ^]^ Moreover, Cu incorporation within the inner core of the alloy NC shortens the bond lengths, leading to a 5.93% reduction in the distance between the two capping Se atoms compared to the Cu‐free Au core (Figure 1E,F), ultimately yielding a more compact metal core with stronger interactions. This structural change effectively alters the electronic structure and photophysical performance of the NC.^[^ ^48^, ^49^, ^50^ ^]^

### 2.2 NIR‐II PL and Photothermal Conversion of Cu_x_Au_61‐x_ NCs

**Figure 2:**
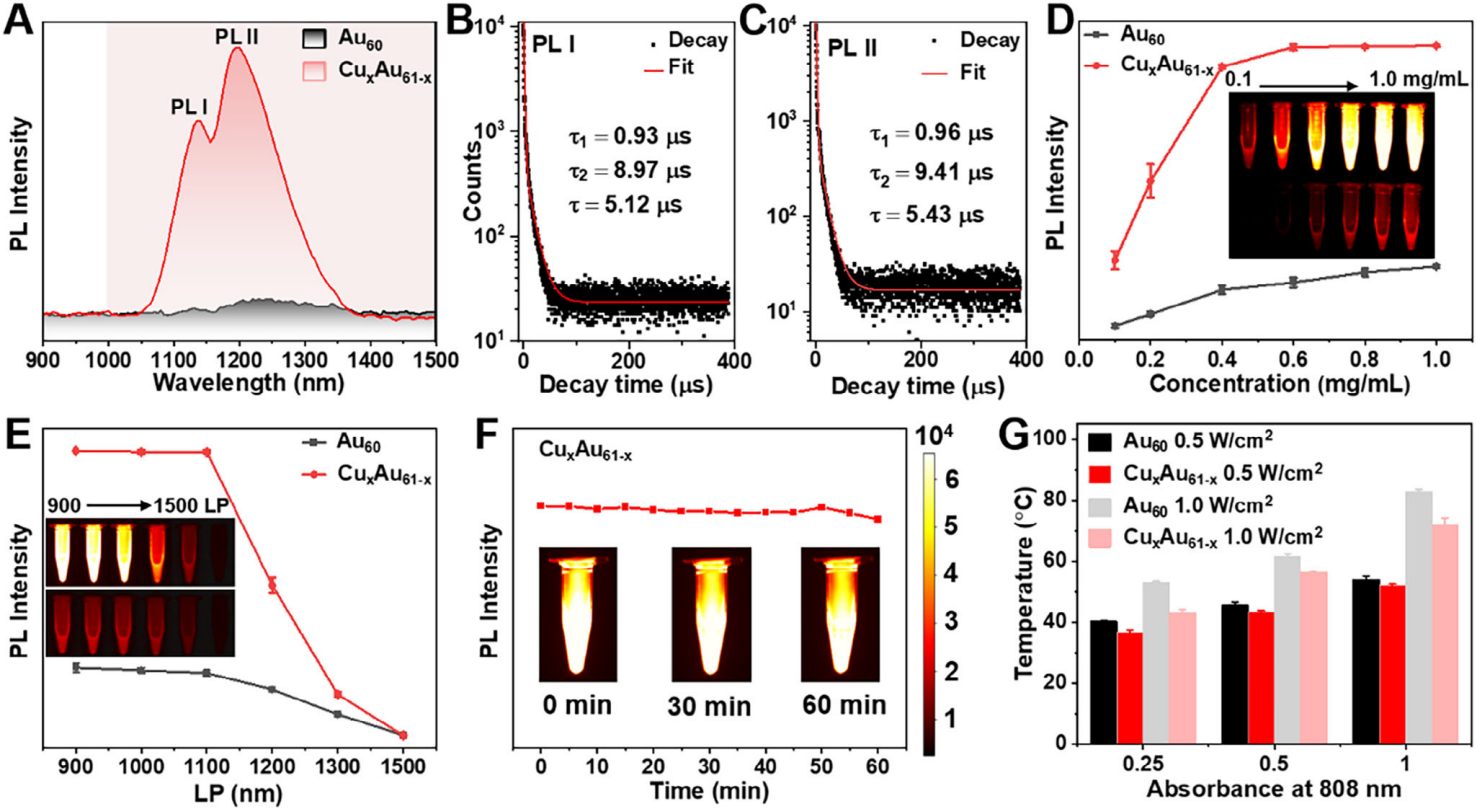
PL and photothermal conversion of Cu_x_Au_61‐x_ NCs. A) NIR‐II emission spectra of Cu_x_Au_61‐x_ compared to Au_60_. The PL decay traces for B) PL I and C) PL II emission of Cu_x_Au_61‐x_ in DMSO. D) PL intensity of Cu_x_Au_61‐x_ and Au_60_ at different concentrations, inset: the corresponding NIR‐II images (900 nm LP filter). (E) PL intensity of Cu_x_Au_61‐x_ and Au_60_ (1.0 mg mL^−1^) with different LP filters, inset: the corresponding NIR‐II images. F) Photostability of Cu_x_Au_61‐x_ excited with an 808 nm laser for 60 min (0.2 W cm^−2^), inset: the corresponding NIR‐II images. G) Temperature variation of Au_60_ and Cu_x_Au_61‐x_ NCs in DMSO by 808 nm irradiation for 10 min.

Doping Cu into the inner part of the NC greatly enhanced the NIR‐II luminescence of Cu_x_Au_61‐x_ (1.0 mg mL^−1^), which exhibited two distinct emission peaks centered at 1138 nm (PL I) and 1197 nm (PL II), while the emission of Au_60_ was barely observable under the same excitation condition (**Figure** **2**A). Both PL I and PL II show similar character—having two lifetime components of ≈0.9 and ≈9 µs in dimethyl sulfoxide (DMSO) (Figure 2B,C). The microsecond lifetimes indicate that PL II is emission from the triplet state, whereas PL I can be assigned to thermally‐assisted delayed luminescence due to reverse intersystem crossing.^[^ ^13^ ^]^ The relative quantum yield of Cu_x_Au_61‐x_ was determined to be 0.75% by using [Au_25_(SC_2_H_4_Ph)_5_(PPh_3_)_10_X_2_]^2+^ (X = Cl/Br, quantum yield = 8%) as a reference (Figure S2, Supporting Information).^[^ ^51^ ^]^

Motivated by the enhanced NIR‐II emission of Cu_x_Au_61‐x_, we performed PL imaging and intensity at different concentrations by an NIR‐II imaging system (Figure 2D). The NIR‐II luminescence signals gradually enhance with the increase of concentration under a 900 nm long‐pass (LP) filter and reach the brightness saturation of the detector at ≈0.5 mg mL^−1^ for Cu_x_Au_61‐x_. The NIR‐II PL of Cu_x_Au_61‐x_ (1.0 mg mL^−1^) was also investigated using LP filters of different cutoff wavelengths (Figure 2E), and the PL signal significantly dropped when using a 1200 nm LP filter, consistent with the NIR‐II emission peak of Cu_x_Au_61‐x_. Of note, under identical conditions, the PL of undoped Au_60_ was generally weak under the imaging system.

The photostability of Cu_x_Au_61‐x_, a critical challenge for luminescence probes in bioimaging applications,^[^ ^52^, ^53^ ^]^ was assessed via excitation at 808 nm, with a power density of 0.2 W cm^−^^2^ for 60 min (Figure 2F). No obvious photobleaching was observed during the continuous irradiation, and the initial brightness was maintained throughout the time, suggesting the excellent photostability of Cu_x_Au_61‐x_ (1.0 mg mL^−1^) in DMSO solution. The long‐term stability test of Cu_x_Au_61‐x_ also showed no obvious decrease in PL intensity when stored in DMSO solution at room temperature for one week (Figure S3, Supporting Information), nor any discernible change in UV–vis spectra (Figure S4, Supporting Information). These results confirmed that Cu_x_Au_61‐x_ holds great promise in applications such as NIR‐II bioimaging.

Beyond NIR‐II PL, photothermal conversion has been observed recently in anisotropic NCs,^[^ ^54^ ^]^ and such a property is also found in Cu_x_Au_61‐x_ and Au_60_ NCs (dissolved in DMSO). Both NCs have an oblate structure and strong absorption at the common irradiation wavelength of 808 nm for photothermal evaluation. To more effectively investigate the differences in temperature change, the absorbance was employed as the variable, rather than the concentration. The temperature change was recorded using an infrared camera. As shown in Figure 2G and Figure S5 (Supporting Information), with a laser power density of 0.5 W cm^−2^ for 10 min, the temperature of the DMSO solution increased to 36.9, 43.4, and 50.8 °C for the Cu_x_Au_61‐x_ NC at the absorbance of 0.25, 0.5, and 1.0, respectively. While the corresponding maximum temperature of Au_60_ NC was found to be 40.3, 45.8, and 54.1 °C, which is slightly higher than those of Cu_x_Au_61‐x_ at the same absorbance. The stronger NIR‐II luminescence of Cu_x_Au_61‐x_ may be the primary factor to this phenomenon. At even higher laser power density of 1 W cm^−2^ (continuously for 10 min), the temperature variation of Cu_x_Au_61‐x_ and Au_60_ NCs (absorbance at 808 nm = 1) was found to be as high as 71.9 and 82.9 °C, respectively. These findings demonstrate that both Cu_x_Au_61‐x_ and Au_60_ NCs can serve as excellent candidates for photothermal agents, but only Cu_x_Au_61‐x_ possesses both strong NIR‐II luminescence and photothermal functionalities.

### 2.3 Excited‐State Dynamics of Cu_x_Au_61‐x_

**Figure 3:**
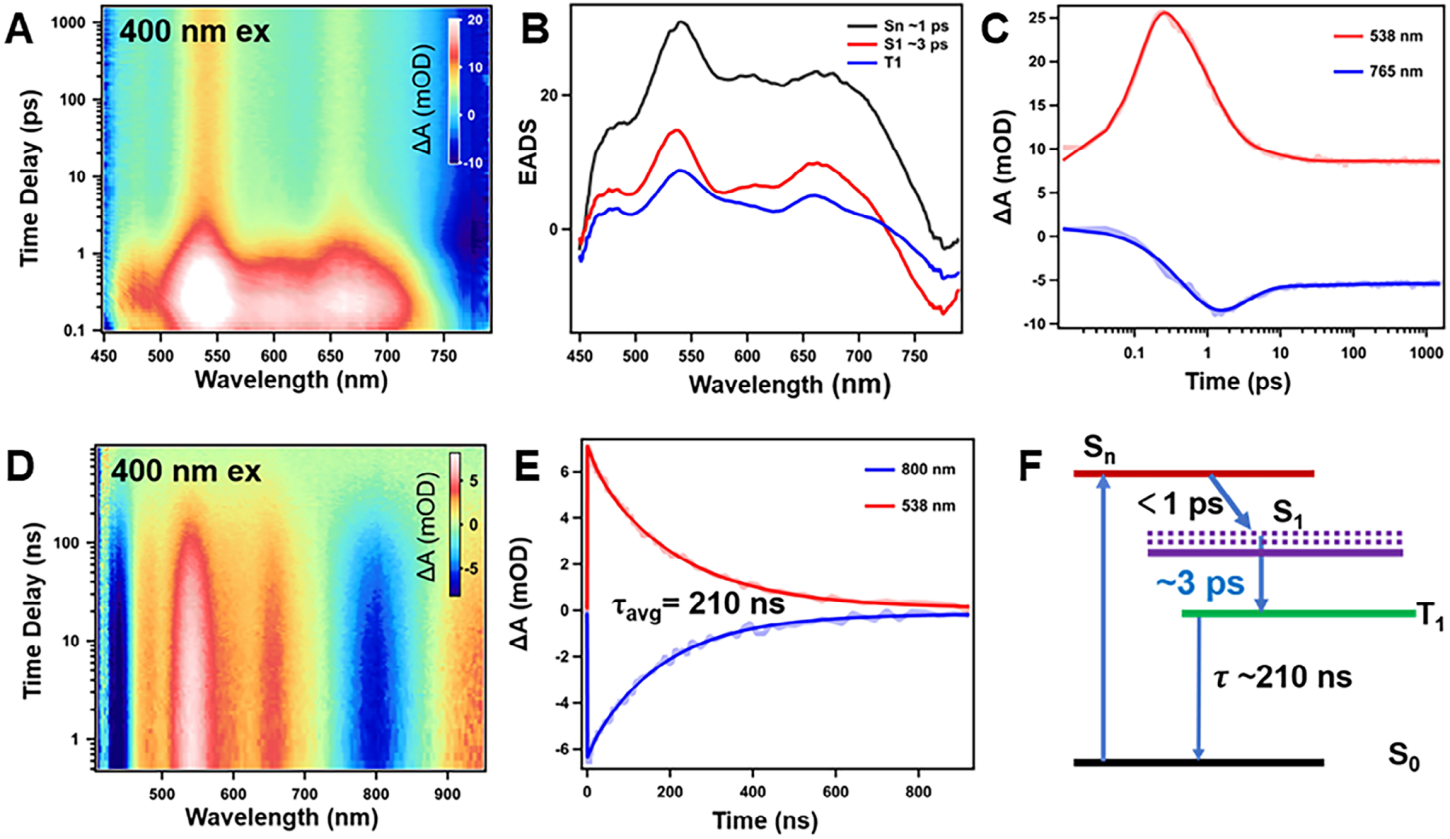
Excited‐State Dynamics of Cu_x_Au_61‐x_. A) fs‐TA data map of Cu_x_Au_61‐x_ in DMSO with 400 nm excitation. B) Global analysis results derived from the fs‐TA of Cu_x_Au_61‐x_. C) fs‐TA kinetic traces and corresponding fits at selected probe wavelengths. D) ns‐TA data map of Cu_x_Au_61‐x_ in DMSO with 400 nm excitation. E) ns‐TA kinetic traces and corresponding fits at selected probe wavelengths. F) Diagram of the excited‐state deactivation mechanism of Cu_x_Au_61‐x_.

To understand the photophysics of Cu_x_Au_61‐x_, we probed its excited‐state dynamics by transient absorption (TA) spectroscopy.^[^ ^55^ ^]^ The femtosecond transient absorption (fs‐TA) spectra of Cu_x_Au_61‐x_ in DMSO under 400 nm excitation revealed initial TA spectra consisting of ground‐state bleaching (GSB, negative) signals at 450 and 800 nm, along with excited‐state absorption (ESA, positive) bands at 550, 600, and 660 nm (**Figure** **3**A). Between 0 and 3 ps, a rapid relaxation in the 550–700 nm ESA bands was observed. As the time delay increased to 40 ps, all ESA bands gradually decreased, and the TA spectra of Cu_x_Au_61‐x_ remained nearly unchanged from 40 to 1600 ps. The fast decay of the ESA features, followed by the slower decay, suggests that the interconversion (IC) and intersystem crossing (ISC) processes should be responsible for these behaviors.

Global fitting of the fs‐TA data indicates that the dynamics across all wavelengths can be well described by a model consisting of an ultrafast relaxation (1 ps), a picosecond relaxation (3 ps), and a long‐lived state (>>1 ns) (Figure 3B,C). To accurately characterize the long‐lived species, we further performed nanosecond transient absorption spectroscopy (ns‐TA), which revealed a lifetime of ≈210 ns (Figure 3D,E). Within the ns‐TA measurement window, no spectral evolution was observed except for a monotonic attenuation of the signal. To elucidate the excited‐state processes and extract the ISC kinetics of Cu_x_Au_61‐x_, a simplified excited‐state deactivation mechanism was proposed based on the fs‐TA results (Figure 3F). To confirm the origin of the second relaxation process (3 ps), the excited‐state dynamics of Cu_x_Au_61‐x_ were compared in the low‐polar solvent dichloromethane (DCM) (Figure S6, Supporting Information). It was found that the time constant of the second process is regulated by solvent polarity: in less polar solvents, the second decay component slows down significantly because the S_1_ energy level is sensitive to solvent polarity. In highly polar solvents, the S_1_ energy level drops, reducing the singlet‐triplet energy level difference (Δ*E*_ST_) and thereby accelerating the ISC process.

For a comparison with Cu_x_Au_61‐x_, we also probed the excited‐state dynamics of the undoped Au_60_ in DMSO. The fs‐TA spectra at the initial delay times (Figure S7, Supporting Information) displayed characteristic absorption peaks comprising broad ESA and GSB (≈850 nm). Subsequently, all ESA bands’ intensities decayed rapidly, showing a similar pattern to that of Cu_x_Au_61‐x_. However, the time constant of the ISC process, associated with solvation stabilization relaxation, was determined to be 12 ps in DMSO (with an ISC rate constant of 45 ps in less polar solvent DCM, Figure S8, Supporting Information), which is much slower than that of Cu_x_Au_61‐x_. The faster ISC rate in Cu_x_Au_61‐x_ (3 ps) compared to Au_60_ (12 ps), both in DMSO, indicates an enhanced spin‐orbit coupling and a smaller singlet‐to‐triplet energy gap after Cu doping.^[^ ^48^ ^]^ We rationalize that the central Cu insertion—which integrates the penta‐icosahedral structure into a more compact one (see Figure 1E,F) with more bonds, may benefit spin‐orbit coupling compared to the undoped Au_60_ NC. The ns‐TA measurements revealed that the average TA lifetime of the Au_60_ NC is ≈100 ns (Figure S7D–F, Supporting Information), significantly shorter than the lifetime observed in the alloy NC. The longer excited state lifetime in Cu_x_Au_61‐x_ compared to Au_60_ agrees with the NIR‐II PL results.^[^ ^56^ ^]^

### 2.4 Photodynamic Property of Cu_x_Au_61‐x_

**Figure 4:**
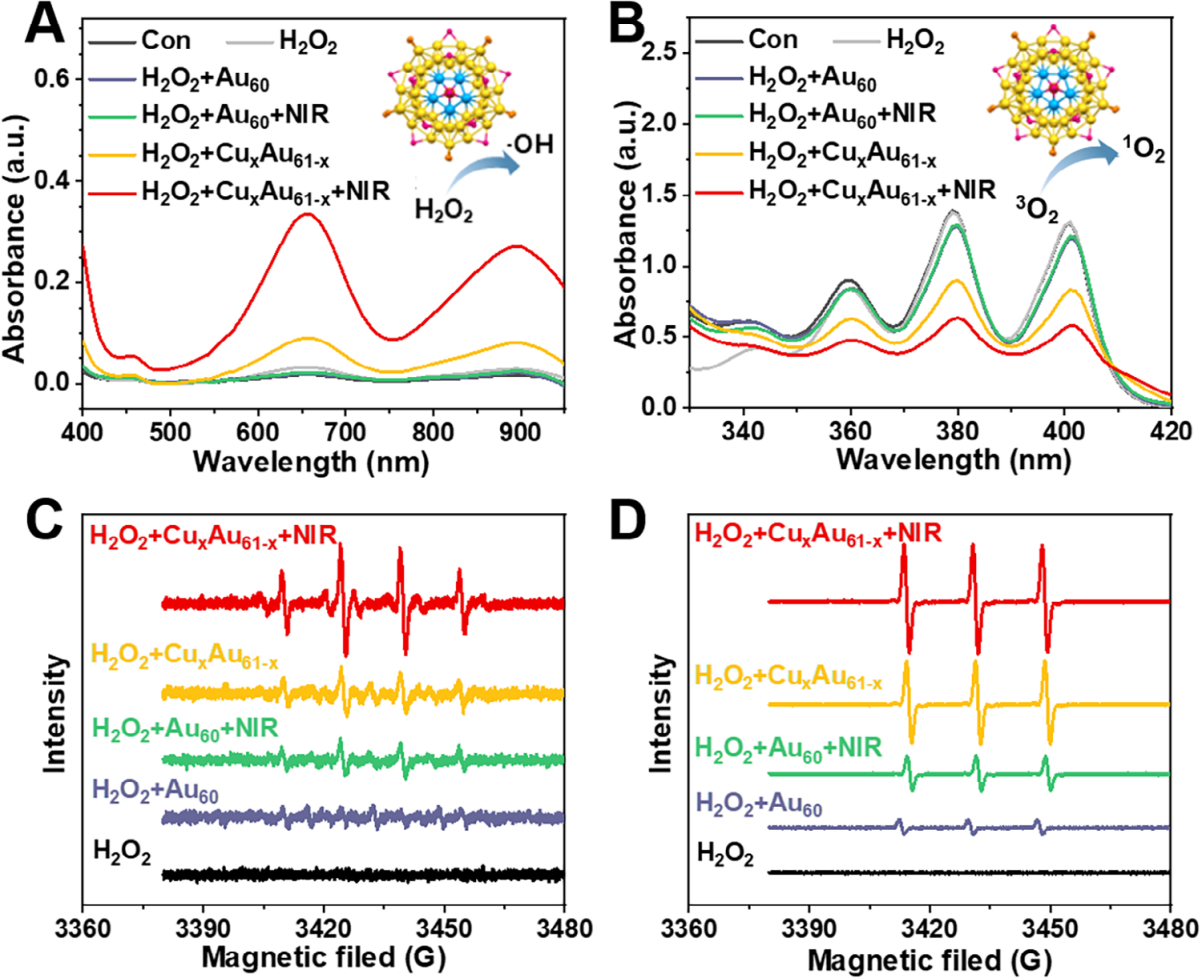
Photodynamic property of Cu_x_Au_61‐x_. UV–vis absorption spectra of A) TMB and B) ABDA oxidation under different conditions. ESR spectra of C) the ∙OH generation using DMPO and D) the ^1^O_2_ generation using TEMP under different treatment conditions.

As the incorporation of Cu atoms can enhance enzyme‐like activity,^[^ ^57^, ^58^, ^59^ ^]^ we evaluated the potential enzyme‐like behavior of Cu_x_Au_61‐x_. The NCs were dissolved in DMSO and dispersed in deionized (DI) water for subsequent experiments in the presence of H_2_O_2_ (1 mM) (**Figure** **4**A). To assess peroxidase‐like activity, 3,3′,5,5′‐tetramethylbenzidine (TMB) was used as a probe.^[^ ^60^ ^]^ UV–vis spectra revealed the characteristic peak of oxidized TMB (ox‐TMB) at 652 nm in the presence of Cu_x_Au_61‐x_ and H_2_O_2_. Notably, when the solution was further exposed to 808 nm laser irradiation at a power density of 1 W cm^−2^ for 10 min, the intensity of the ox‐TMB peak was significantly enhanced (Figure 4A). In contrast, the combination of undoped Au_60_ NC and H_2_O_2_ showed negligible changes even under laser irradiation, indicating that Cu_x_Au_61‐x_ possesses a much higher peroxidase‐like activity, efficiently catalyzing the generation of hydroxyl radicals (∙OH), particularly under NIR laser stimulation. The generation of singlet oxygen (^1^O_2_) was detected by adding 9,10‐anthracene bis(methylene) dimalonic acid (ABDA).^[^ ^61^ ^]^ As shown in Figure 4B, the intensity of ABDA's characteristic peak at 400 nm progressively decreased upon the addition of Cu_x_Au_61‐x_ in the presence of H_2_O_2_, suggesting effective ^1^O_2_ production, and this effect was further amplified under laser irradiation. In contrast, the Au_60_ NC showed negligible generation of ^1^O_2_.

Subsequently, the generation of ROS was further confirmed by electron spin resonance (ESR) spectroscopy, using 5,5‐dimethyl‐1‐pyrroline *N*‐oxide (DMPO) and 2,2,6,6‐tetramethylpiperidine (TEMP) as spin‐trapping agents for ∙OH and ^1^O_2_, respectively.^[^ ^62^ ^]^ Characteristic EPR signals with intensity ratios of 1:2:2:1 (for DMPO/∙OH) and 1:1:1 (for TEMP/^1^O_2_) were observed for the combination of H_2_O_2_, Cu_x_Au_61‐x,_ and NIR radiation. These results further validate that Cu doping markedly improves the catalytic performance for ROS regeneration, underscoring its potential antitumor applications (vide infra). The photodynamic activity of Cu_x_Au_61‐x_ further suggests that its NIR‐II emission originates from a phosphorescence mechanism, as the triplet excited state can transfer energy to ^3^O_2_, generating singlet oxygen ^1^O_2_.

### 2.5 Characterization of Aqueous Cu_x_Au_61‐x_@SiO_2_–FA

**Figure 5:**
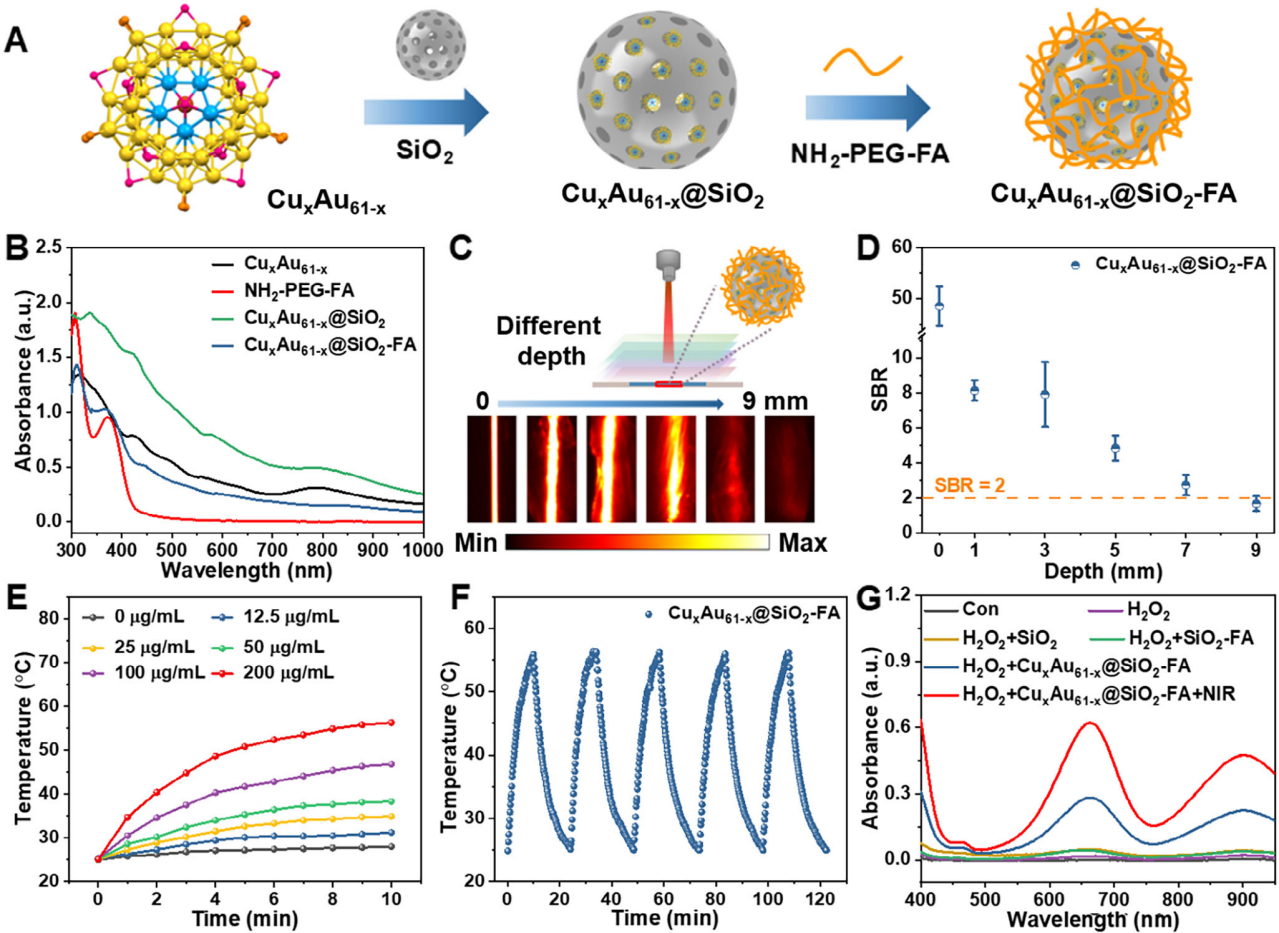
Characterization of Cu_x_Au_61‐x_@SiO_2_–FA. A) Synthetic scheme of the Cu_x_Au_61‐x_@SiO_2_–FA nanoplatform. B) UV‐vis absorption spectra of Cu_x_Au_61‐x_ before and after loading and SiO_2_ modification. C) Schematic illustration of the penetration depth assay for Cu_x_Au_61‐x_@SiO_2_–FA (200 µg mL^−1^) and in vitro NIR‐II PL images of capillaries covered with chicken breast at varying depths under irradiation. D) SBR of Cu_x_Au_61‐x_@SiO_2_–FA at different penetration depths. E) Temperature curves of Cu_x_Au_61‐x_@SiO_2_–FA dispersions at different concentrations under 10 min of irradiation (1.0 W cm^−2^). F) Heating and cooling profiles of 200 µg mL^−1^ Cu_x_Au_61‐x_@SiO_2_–FA dispersions over 5 repeated ON/OFF irradiation cycles. G) UV–vis absorption spectra of TMB under various treatment conditions. Irradiation: 808 nm, 1.0 W cm^−2^.

Given the triple‐functionality of Cu_x_Au_61‐x_ —NIR‐II PL, photothermal conversion, and photodynamic activity, we further developed Cu_x_Au_61‐x_@SiO_2_ hybrids for potential imaging‐guided tumor therapy. Mesoporous SiO_2_, which was recognized as a biocompatible carrier,^[^ ^63^ ^]^ was synthesized to encapsulate the Cu_x_Au_61‐x_ NCs (**Figure** **5**A; Figure S9, Supporting Information). The resulting Cu_x_Au_61‐x_@SiO_2_ displayed the same characteristic absorption peaks as the free Cu_x_Au_61‐x_ NCs (Figure 5B, green line), indicating the successful loading of NCs into the mesoporous SiO_2_. This was further proved by energy dispersive spectroscopy (EDS), transmission electron microscopy (TEM), and element mapping (Figure S10, Supporting Information), which demonstrated that some ≈2 nm NCs with uniformly distributed Au, Cu elements were observed within the SiO_2_ matrix. The Cu_x_Au_61‐x_@SiO_2_ also demonstrates excellent photostability in water, as both PL intensity and absorbance remained steady after 7 days, and no obvious change was observed under continuous irradiation with an 808 nm laser (0.2 W cm^−^^2^) for 60 min (Figure S11, Supporting Information). To enhance tumor targeting, the surface of Cu_x_Au_61‐x_@SiO_2_ was further functionalized with NH_2_–PEG(polymer poly(ethylene glycol))–FA (folic acid) polymer,^[^ ^64^, ^65^ ^]^ yielding Cu_x_Au_61‐x_@SiO_2_–FA (Figure 5A). After modification, a characteristic peak at ≈370 nm, corresponding to NH_2_–PEG–FA, was observed in the Cu_x_Au_61‐x_@SiO_2_–FA, which also exhibited a less negative zeta potential compared to Cu_x_Au_61‐x_@SiO_2_ due to polymer grafting (Figure S12, Supporting Information). Fourier transform infrared (FT‐IR) spectra of Cu_x_Au_61‐x_@SiO_2_–FA also confirmed successful surface modification, showing characteristic vibrational peaks of –CH_2_ (≈2900 cm^−1^) and C–O–C (≈1100 cm^−1^) stretching modes of NH_2_–PEG–FA (Figure S13, Supporting Information).^[^ ^66^ ^]^

The Cu_x_Au_61‐x_@SiO_2_–FA nanoplatform showed broad NIR‐II emission ranging from 1045 to 1365 nm (Figure S14, Supporting Information), consistent with the emission of free Cu_x_Au_61‐x_ NCs. For the evaluation of penetration depth and SBR, a capillary tube filled with Cu_x_Au_61‐x_@SiO_2_–FA (200 µg mL^−1^) was imaged through tissues of varying thicknesses (Figure 5C). Although NIR‐II photon intensity was gradually attenuated due to tissue absorption and scattering as the penetration depth increased, the signal remained detectable at a penetration depth up to 9.00 mm. When the SBR decreased to ≈2, the effective imaging depth was slightly reduced to ≈7.0 mm (Figure 5D). Based on the cross‐sectional line profiles (Figure S15, Supporting Information), it can be concluded that Cu_x_Au_61‐x_@SiO_2_–FA exhibits excellent deep penetration capability with high imaging quality.

Aqueous solutions of Cu_x_Au_61‐x_@SiO_2_–FA were subjected to irradiation (808 nm, 1.0 W cm^−2^), and the temperature change was recorded. After 10 min of irradiation, the maximum temperature reached 31.2, 34.9, 38.3, 46.8, and 56.3 °C at Cu_x_Au_61‐x_@SiO_2_–FA concentrations of 12.5, 25, 50, 100, and 200 µg mL^−1^, respectively (Figure 5E; Figure S16, Supporting Information), whereas no noticeable changes were observed in the temperature of both DI water and the aqueous solutions of SiO_2_‐FA (Figure S17, Supporting Information), confirming the good photothermal performance of Cu_x_Au_61‐x_@SiO_2_–FA in aqueous solutions. Based on the temperature changes observed at different concentrations under laser power density of 0.5 and 2.0 W cm^−2^ (Figure S18, Supporting Information), the optimal condition for effective PTT was determined to be 1 W cm^−2^ and 200 µg mL^−1^. Notably, the Cu_x_Au_61‐x_@SiO_2_–FA exhibited exceptional photothermal stability, maintaining consistent performance over five laser on/off cycles (Figure 5F). Finally, the ROS generation capacity of Cu_x_Au_61‐x_@SiO_2_–FA (200 µg mL^−1^) was assessed. The characteristic peak of ox‐TMB at 652 nm was observed in the presence of H_2_O_2_ and Cu_x_Au_61‐x_@SiO_2_–FA (Figure 5G), with the signal further enhanced upon 808 nm irradiation with the laser power density of 1.0 W cm^−2^, whereas this phenomenon was not observed in the presence of H_2_O_2_ and SiO_2_ (or SiO_2_–FA). All these results confirm that Cu_x_Au_61‐x_@SiO_2_–FA exhibits excellent PTT and PDT performance in aqueous solution, supporting its potential for effective antitumor applications.

### 2.6 In Vitro Antitumor Activity of Aqueous Cu_x_Au_61‐x_@SiO_2_–FA

**Figure 6:**
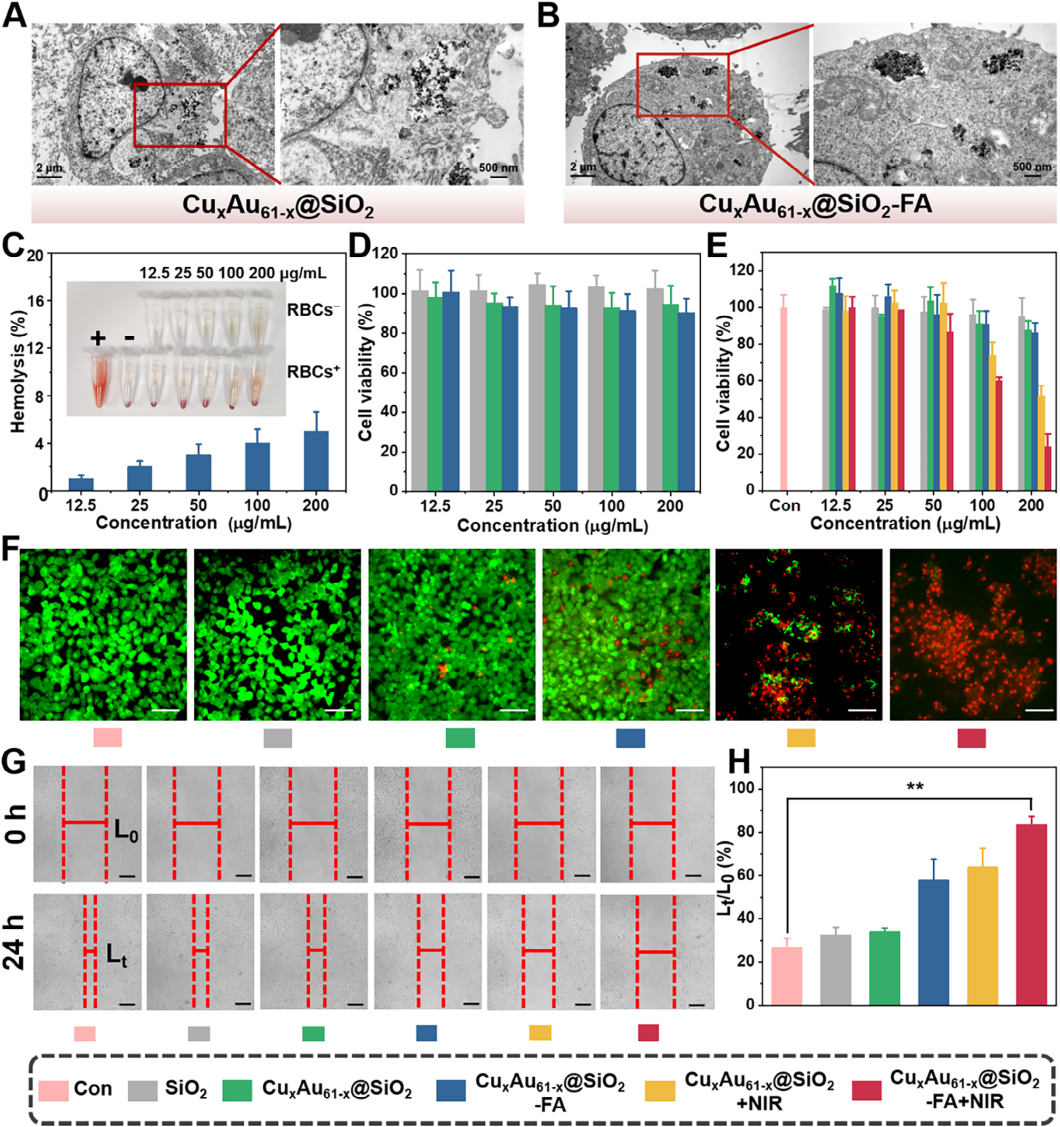
In vitro antitumor activity of Cu_x_Au_61‐x_@SiO_2_–FA. Bio‐TEM images of CAL27 cells incubated with A) Cu_x_Au_61‐x_@SiO_2_ and B) Cu_x_Au_61‐x_@SiO_2_–FA for 24 h. C) Hemolysis of the Cu_x_Au_61‐x_@SiO_2_–FA at different concentrations (*n* = 3). D) Viability of 3T3 cells after 24 h incubation with different treatments without irradiation (*n* = 4). E) Viability of CAL27 cells after different treatments with or without irradiation (808 nm, 1.0 W/cm^2^) (*n* = 4). F) Fluorescence microscopy images of CAL27 cells after different treatments (scale bar = 100 µm). G) Scratch assay images of CAL27 cells after different treatments (scale bar = 200 µm). H) Quantitative analysis of scratch results (*n* = 3).

The tumor cell targeting and antitumor efficacy of Cu_x_Au_61‐x_@SiO_2_‐FA was initially evaluated at the cellular level. First, the endocytosis efficiency of the materials was evaluated by bio‐TEM images. Cu_x_Au_61‐x_@SiO_2_ nanoparticles can be internalized by tumor cells and distributed within cytoplasmic vesicles (**Figure** **6**A), and even more Cu_x_Au_61‐x_@SiO_2_‐FA nanoparticles were accumulated in the cytoplasmic vesicles (Figure 6B), which can be ascribed to the surface modification by the tumor targeted polymer (NH_2_–PEG–FA). Based on effective endocytosis of this material, intracellular ROS generation was further evaluated using the ROS probe 2′,7′‐dichlorofluorescein diacetate (DCFH‐DA) staining method. Cu_x_Au_61‐x_@SiO_2_–FA group exhibited bright green luminescence (Figure S19, Supporting Information), which was significantly intense than the control and SiO_2_ and Cu_x_Au_61‐x_@SiO_2_ groups (Figure S20, Supporting Information), indicating effective ROS generation of Cu_x_Au_61‐x_@SiO_2_–FA and tumor targeting. Notably, more pronounced green luminescence was observed in the Cu_x_Au_61‐x_@SiO_2_–FA +NIR (together with NIR irradiation), indicating that the generation of ROS was markedly enhanced by PTT and PDT.

The good biocompatibility of Cu_x_Au_61‐x_@SiO_2_–FA was first evaluated using a standard hemolysis test in which the hemolysis rates remained below the 5% threshold even at the high concentration of 200 µg mL^−1^ (Figure 6C). Biosafety and antitumor activity were further assessed at the cellular level using the (methylthiazolyldiphenyl‐tetrazolium bromide) MTT assay. After incubating the materials with normal mouse fibroblast 3T3 cells at different concentrations for 24 h, cell viability in all groups remained ≈90%, even at 200 µg mL^−1^, indicating low cytotoxicity (Figure 6D). In contrast, the viability of CAL27 tumor cells progressively declined with increasing concentrations of Cu_x_Au_61‐x_@SiO_2_–FA, due to elevated H_2_O_2_ levels and acidic pH conditions of the tumor microenvironment (TME). Significantly, upon 808 nm irradiation (1 W cm^−2^ for 10 min), the inhibition of tumor cells by Cu_x_Au_61‐x_@SiO_2_–FA was dramatically enhanced, achieving a ≈76% cell death at 200 µg mL^−1^ (Figure 6E, red bars). The enhanced effect can be attributed to the increased ROS generation and elevated temperature induced by NIR irradiation, consistent with the photothermal and photodynamic activities of Cu_x_Au_61‐x_. Further validation was given by live/dead staining assay of CAL27 cells using Calcein‐AM/propidium iodide (PI) double‐staining. Bright green fluorescence was observed in all groups without irradiation (Figure 6F; Figure S21, Supporting Information), indicating favorable biosafety. However, following 10 min of 808 nm irradiation at 1.0 W cm^−2^, a significant increase in red luminescence was noticed, particularly in the Cu_x_Au_61‐x_@SiO_2_–FA +NIR group (Figure 6F, rightmost panel), confirming that the antitumor effect of Cu_x_Au_61‐x_@SiO_2_–FA is mediated by its excellent photothermal and photodynamic activities.

Cell scratch assays were conducted to evaluate the inhibitory effect of the materials on tumor cell migration. By comparing the scratch areas before and after 24 h, it was observed that the control group showed nearly complete wound healing. In contrast, the Cu_x_Au_61‐x_@SiO_2_–FA +NIR group demonstrated a remarkable CAL27 cell inhibition rate of 83.4% following treatment with 808 nm irradiation (Figure 6G,H). Collectively, these results consistently suggest that Cu_x_Au_61‐x_@SiO_2_–FA not only exhibits high biosafety but also efficiently induces apoptosis and inhibits tumor cell migration and metastasis under laser irradiation.

### 2.7 In Vivo Imaging and Antitumor Activity of Cu_x_Au_61‐x_@SiO_2_–FA

Leveraging the enhanced NIR‐II PL intensity of Cu_x_Au_61‐x_ NCs, accurate treatment and noninvasive monitoring can be achieved through NIR‐II imaging by tracking the biodistribution of Cu_x_Au_61‐x_@SiO_2_–FA in vivo. To evaluate this, Cu_x_Au_61‐x_@SiO_2_ and Cu_x_Au_61‐x_@SiO_2_–FA were intravenously injected into CAL27 tumor‐bearing BALB/c mice via the tail vein (Figure S22A, Supporting Information). Real‐time tumor retention was monitored at different time points (0, 2, 4, 8, 12, and 24 h) using whole‐body NIR‐II imaging with a 900 LP filter (Figure S22B, Supporting Information). Over time, PL intensity in both groups initially increased and then decreased. Notably, the Cu_x_Au_61‐x_@SiO_2_–FA group reached peak PL intensity at 4 h post‐injection, whereas the Cu_x_Au_61‐x_@SiO_2_ group peaked at 6 h, and the significantly higher peak PL intensity in the FA‐functionalized group highlights its superior tumor‐targeting efficacy (Figure S22C, Supporting Information). At 24 h post‐injection, the mice were sacrificed, and major organs and tumors were harvested for subsequent imaging (Figure S22D, Supporting Information) and semi‐quantitative analysis (Figure S22E, Supporting Information). Although both nanomaterials showed predominant metabolization via hepatic and splenic accumulation, Cu_x_Au_61‐x_@SiO_2_–FA displayed higher concentrations at tumor sites compared to its non‐targeted counterpart (Figure S22D, Supporting Information), indicating that tumor targeting not only enhanced the accumulation of nanomaterials at tumor sites but also significantly reduced systemic transit time. Based on these findings, 4 h post‐injection is identified as the optimal time point for initiating phototherapy with Cu_x_Au_61‐x_@SiO_2_–FA.

**Figure 7:**
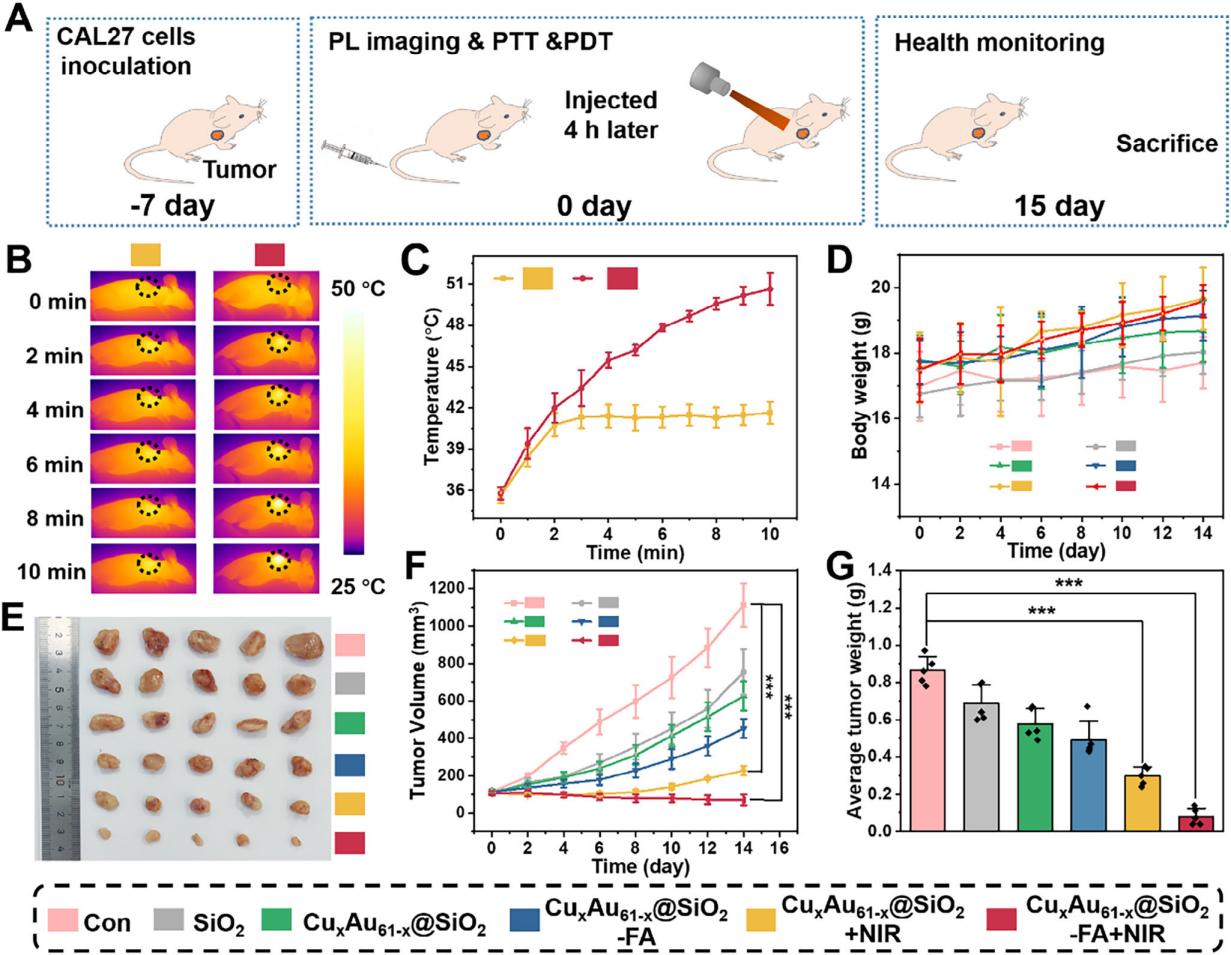
In vivo antitumor activity of Cu_x_Au_61‐x_@SiO_2_–FA. A) Schematic illustration of the antitumor treatment process. B) Infrared thermal images of mice post‐injection of Cu_x_Au_61‐x_@SiO_2_ and Cu_x_Au_61‐x_@SiO_2_–FA under different irradiation durations. C) Temperature variation curves of tumor regions during irradiation. D) Body weight curves of mice from different treatment groups. E) Tumor photographs in different groups. F) Tumor volume curves over time following different treatments. G) Tumor weight at the end of different treatments. (mean ± SD, *n* = 5, *P < 0.05, ^**^ *P* < 0.01, ^***^ *P* < 0.001). Radiation: 808 nm, 1.0 W cm^−2^.

Encouraged by the in vitro performance of Cu_x_Au_61‐x_@SiO_2_–FA, we further explored its therapeutic efficacy in vivo. CAL27 tumor‐bearing BALB/c nude mice models were established, and after one week, the tumor volume reached ≈100 mm^3^. The mice were randomly divided into treatment groups (*n* = 5) to monitor tumor progression throughout the therapeutic process. The nanomaterial (10 mg kg^−1^) was injected into the tail vein, and NIR‐II PL imaging demonstrated that the optimal time for phototherapy initiation was 4 h post‐injection. Accordingly, 808 nm irradiation was applied at the 4th h after injection, and temperature changes at the tumor site were recorded with an NIR camera throughout the irradiation period (**Figure** **7**A). As irradiation time increased, the tumor site temperature progressively rose (Figure 7B,C; Figure S23, Supporting Information). At 10 minutes of irradiation, the temperatures at the tumor sites in the Cu_x_Au_61‐x_@SiO_2_ +NIR and Cu_x_Au_61‐x_@SiO_2_‐FA +NIR groups reached 41.6 and 50.6 °C, respectively. In the Cu_x_Au_61‐x_@SiO_2_‐FA +NIR group, the combined effects of elevated temperature and ROS generation led to complete tumor tissue ablation.

During the treatment period, body weight and tumor volume of all mice were recorded every 2 days. Over the course of 14 days, mice in all groups exhibited steady weight gain (Figure 7D), indicating that the injection of nanomaterials did not affect the diet of mice or induce systemic toxicity. Photographic documentations during the treatment period revealed complete ablation of the tumor in the Cu_x_Au_61‐x_@SiO_2_–FA +NIR group, leaving a black scar that resolved by day 14. In contrast, tumors in the other groups continued to grow to varying degrees, particularly in the control group, where pronounced subcutaneous masses were observed (Figure S24, Supporting Information). At the end of the treatment, tumors were excised, weighed, and photographed (Figure 7E,F,G). The results illustrated that the Cu_x_Au_61‐x_@SiO_2_–FA +NIR group achieved a high tumor inhibition ratio of ≈90% under 808 nm irradiation, attributed to the synergistic PTT and PDT effects.

To further evaluate therapeutic efficacy, histological analysis was performed. Hematoxylin and eosin (H&E) staining as well as TdT‐mediated dUTP Nick‐End labeling (TUNEL) staining revealed significant apoptosis and necrosis in tumor tissues for the Cu_x_Au_61‐x_@SiO_2_–FA +NIR group (Figure S25A,B, Supporting Information). In addition, Ki67 immunohistochemical staining showed a substantial reduction in nuclear antigen expression, indicating significantly suppressed tumor cell proliferation (Figure S25C, Supporting Information). Furthermore, major organs (heart, liver, spleen, lung, and kidney) of mice from all groups were collected for H&E analysis to evaluate the in vivo biosafety of nanomaterials, revealing no obvious pathological changes (Figure S26, Supporting Information). Furthermore, blood biochemistry analysis showed no appreciable abnormalities across groups, once again confirming the excellent biocompatibility of Cu_x_Au_61‐x_@SiO_2_–FA (Figure S27, Supporting Information).

## 3 Conclusion

In summary, an atomically precise Cu_x_Au_61‐x_ NC is obtained using the penta‐icosahedral Au_60_ NC as a template via reaction with Ph_3_P–Cu(I)–Br complex. The doped Cu atoms occupy the inner part of the NC, with an additional Cu atom in the center, which leads to a more compact structure and significantly enhances NIR‐II emission and ROS generation compared to the undoped Au_60_. Additionally, Cu_x_Au_61‐x_ exhibits rapid photothermal conversion, reaching ≈70 °C within 10 min under 808 nm irradiation, making it a triple‐functional NC that is especially suitable for biomedical applications. A multifunctional, water‐dispersible Cu_x_Au_61‐x_@SiO_2_–FA nanoplatform is further constructed and evaluated both in vitro and in vivo. The nanoplatform demonstrated excellent biocompatibility and outstanding performance in NIR‐II luminescence imaging‐guided tumor phototherapy—the NIR‐II PL of Cu_x_Au_61‐x_ enables non‐invasive optimization of treatment timing, whereas its potent photothermal and photodynamic effects ensure efficient tumor ablation.

## 4 Experimental Section

### Synthesis of Cu_x_Au_61‐x_ NC

20 mg Au_60_ NC were dissolved in 10 mL of toluene under vigorous stirring. Then, 10 mg Ph_3_P‐Cu(I)‐Br was added. After 12 h, the peak of Au_60_ NC (≈835 nm) was gradually blue shift to ≈790 nm, indicating the exchange of Cu atoms into the Au_60_ NC. The solution was collected and washed with toluene/*n*‐hexane for 3 times to remove by‐products. Then, the black needle‐like crystals were obtained from toluene/n‐hexane at room temperature. The detailed synthesis methods of Au_60_ and Ph_3_P‐Cu(I)‐Br were provided in the Supporting Information.

### Photothermal Performance

In order to compare the photothermal performance of Au_60_ NC and Cu_x_Au_61‐x_ NC, the two NCs were dissolved in DMSO, and the UV–vis spectral absorbance at 808 nm was determined to be 0.25, 0.5, and 1, respectively. The temperature variation of the solution was recorded by an infrared camera with different power densities (0.5 and 1.0 W cm^−2^). To investigate the photothermal effect of Cu_x_Au_61‐x_@SiO_2_‐FA, different concentrations (0, 12.5, 25, 50, 100, and 200 µg mL^−1^) were irradiated with an 808 nm laser at different power densities (0.5, 1.0, and 2.0 W cm^−2^) for 10 min, respectively. Infrared cameras recorded temperature changes every minute. To further evaluate the photothermal stability of Cu_x_Au_61‐x_@SiO_2_–FA, the solution (200 µg mL^−1^) was heated using 808 nm laser irradiation for 10 min (1.0 W cm^−2^), and then cooled to room temperature with the light source off. The process was repeated five times.

### ROS Detection

ROS generation performance of Au_60_ NC and Cu_x_Au_61‐x_ NC was tested using TMB and ABDA. In detail, 0.2 mg Au_60_ NC and Cu_x_Au_61‐x_ NC were separately dispersed into the 2 mL phosphate buffer (pH 4.0) containing 0.25 mg TMB (or 0.12 mg ABDA) and H_2_O_2_ (1 mm) with/without 808 nm laser irradiation (1.0 W cm^−2^, 10 min). Then, the mixed solution was measured by the UV–vis absorption spectrum.

For ESR measurement of ∙OH, and ^1^O_2_, DMPO and TEMP were added to the same concentration of sample solution, respectively. Then, an ESR spectrometer was used to detect ∙OH, and ^1^O_2_ generation by the sample with or without laser irradiation (1.0 W cm^−2^, 10 min).

### NIR‐II Signal Detection of Au_60_ NC and Cu_x_Au_61‐x_ NC

To compare the NIR‐II emission intensity of Au_60_ NC and Cu_x_Au_61‐x_ NC, two NCs solutions with different concentrations (0.1, 0.2, 0.4, 0.6, 0.8, and 1.0 mg mL^−1^) were configured for detecting the NIR‐II signal with a full‐spectrum fluorescence in vivo imaging system. In addition, 1.0 mg mL^−1^ NC solution was used to detect signal strength changes under different filters (900, 1000, 1100, 1200, 1300, and 1500 LP).

### Cell Line and Culture

The 3T3 and CAL27 cells were cultured in DMEM medium (10% foetal bovine serum (FBS), 1% penicillin‐streptomycin) under 5% CO_2_ at a 37 °C humidification atmosphere. DMEM, FBS, and penicillin‐streptomycin were purchased from meilunbio (Da Lian, China).

### Cell Viability Assays

The cells (3T3, CAL27) were seeded on 96‐well plates at 1*10^4^ cells/well for 24 h. The different concentrations of materials (0, 12.5, 25, 50, 100, and 200 µg mL^−1^) were co‐cultivated with the cells for another 12 h. Then, the CAL27 cells were irradiated by 808 nm laser (1 W cm^−2^, 10 min/well) and incubated for 4 h. 10 µL MTT solution (1 mg mL^−1^) was added to 96‐well plates and further incubated for 4 h. At last, the medium was replaced by 100 µL DMSO and measured with a microplate reader. The 3T3 cells were co‐incubated with different concentrations of materials for 24 h, and MTT was directly added for subsequent detection. Here, 3T3 cells were used as normal cells, and CAL27 cells were introduced as tumor cells.

### In Vitro ROS Assay

DCEH‐DA probe was used to study the generation of intracellular ROS. The CAL27 cells were seeded in a confocal petri dish at 1 × 10^5^ cells/well for 24 h. Then, the materials were added to the dish and incubated for 4 h. The cells were irradiated for 10 min with an 808 nm laser (1.0 W cm^−2^) and washed with PBS. DCFH‐DA was added to the Petri dish for 30 min. After that, the intracellular green fluorescence was recorded by CLSM.

### Live/Dead Cell Staining

CAL27 cells were plated in 24‐well plates at 5 × 10^4^ cells/well for 24 h and performed with different treatments. Then, the Calcein‐AM and propidium iodide (PI) were added to the for the living and dead CAL27 cell staining for 30 min. Subsequently, the cell samples were washed with PBS and captured with fluorescence photographs by a fluorescence microscope.

### Wound‐Healing Assay

CAL27 cells were plated in 6‐well plates at 1 × 10^5^ cells/well, and the culture was continued until the cells were completely confluent monolayer. Subsequently, an aseptic 200 µL pipet tip was used for making a linear scratch on the cell monolayer. The scratched cell residue was gently washed three times with PBS. Then, the cells were incubated with different materials in a free medium and performed to different treatments. After 24 h, the migrated distance in different groups was observed and imaged by a fluorescence microscope.

### Hemolytic Test

Red blood cells (RBCs) were used for the hemolytic test, which were obtained from healthy mice's blood. The RBCs were diluted with PBS at 1: 10 (v/v). Then, the cell dispersions were mixed with PBS (negative control), DI water (positive control), and Cu_x_Au_61‐x_@SiO_2_–FA with different concentrations were incubated at 37 °C for 1 h, respectively. The supernatant solution was separated by centrifugation (3000 rpm, 10 min) and measured by UV–vis spectroscopy. The characteristic absorption peak at 541 nm was observed, and the hemolysis percentage of RBCs was calculated based on the equation:

(1) $$\mathrm{Hemolysis}\;\left(\%\right)=\frac{\left(\mathrm{Ab}s_{\mathrm{RBC}s^{+}}-\mathrm{Ab}s_{\mathrm{RBC}s^{-}}\right)-\mathrm{Ab}s_{\mathrm{negative}}}{\mathrm{Ab}s_{\mathrm{positive}}-\mathrm{Ab}s_{\mathrm{negative}}}$$

### In Vivo NIR‐II Imaging

Before conducting in vivo anti‐tumor experiments, the distribution of materials in mice was first observed. CAL27 cells (5×10^6^ cells in 100 µL PBS) were injected subcutaneously into the right forelimb of BALB/c female nude mice (4‐5 weeks) to establish a mouse tumor model of oral squamous cell carcinoma with CAL27 cells. After a week, the tumor volume was ≈100 mm^3^. The tumor volume was recorded and calculated as follows: tumor volume (mm^3^) = 0.5× (tumor length) × (tumor width)^2^. Mice were injected into the tail vein Cu_x_Au_61‐x_@SiO_2_ or Cu_x_Au_61‐x_@SiO_2_–FA (100 µL, 10 mg kg^−1^) and placed in a full‐spectrum fluorescence in vivo imaging system at different time points (0, 2, 4, 8, 12, and 24 h) for photography. In addition, 24 h after the material was injected, major organs (heart, liver, spleen, lung, and kidney) and tumors of the mice were removed to observe the distribution of the material.

### In Vivo Antitumor Experiments

All mice were purchased from the Center for Laboratory Animal Sciences. All animal experiments were approved by the Ethical Committee of Anhui Medical University (approved number: LLSC20231635). All animal experimental protocols were performed following the guidelines established by the Association of Laboratory Animal Sciences and the Center for Laboratory Animal Sciences at Anhui Medical University. A CAL27 xenograft tumor model was established by the subcutaneous injection of CAL27 cells (5 × 10^6^ cells in 100 µL PBS) in the right forelimb of female BALB/c nude mice (4‐5 weeks). After one week, the tumor volume reached ≈100 mm^3^. The mice were intravenously injected with SiO_2_, Cu_x_Au_61‐x_@SiO_2,_ or Cu_x_Au_61‐x_@SiO_2_–FA.

### Statistical Analysis

Experiment results were expressed as mean ± standard deviation. The statistical significance was analyzed by One‐way ANOVA with Tukey's post‐hoc test. The differences were regarded as statistically significance for the P‐values: ^*^ *P* < 0.05, ^**^ *P* < 0.01, ^***^ *P* <0.001.

[CCDC 2432419 contains the supplementary crystallographic data for this paper. These data can be obtained free of charge from The Cambridge Crystallographic Data Centre via www.ccdc.cam.ac.uk/data_request/cif.]

## Conflict of Interest

The authors declare no conflict of interest.

## Data and materials availability

The data that support the findings of this study are available from the corresponding author upon reasonable request.

## Supporting information



Supporting Information

Supporting Information
