# Supplementary material for: Triple‐Functional CuxAu61‐x Nanoclusters with NIR‐II Photoluminescence, Photothermal and Photodynamic Properties and Their Bio‐Application
Source: Adv Sci (Weinh). 2025 Jul 18;12(39):e09283. doi: 10.1002/advs.202509283 (PMC12533204; doi:10.1002/advs.202509283)
Supplement: Supplementary file 1 — Supporting Information [file ADVS-12-e09283-s001.pdf]

ADVANCED SCIENCE
Open Access

Supporting Information

for *Adv. Sci.*, DOI 10.1002/advs.202509283

Triple-Functional Cu_xAu_{61-x} Nanoclusters with NIR-II Photoluminescence, Photothermal and Photodynamic Properties and Their Bio-Application

Tingting Xu, Jie Kong, Yu Chen, Wenxue Cui, Yucheng Fang, Wei Zhang, Mohan Wang, Meng Zhou, Yingwei Li, Rongchao Jin* and Yongbo Song**

(Supporting information)

Triple-Functional Cu_xAu_{61-x} Nanoclusters with NIR-II Photoluminescence, Photothermal and Photodynamic Properties and Their Bio-application

Tingting Xu,[1†] Jie Kong,[2†] Yu Chen,[1] Wenxue Cui,[1] Yucheng Fang,[1] Wei Zhang,[1] Mohan Wang,[3*] Meng Zhou,[2] Yingwei Li,[4] Rongchao Jin,[4*] and Yongbo Song[1*]

[1]School of Biomedical Engineering, Research and Engineering Center of Biomedical Materials, Anhui Medical University, Hefei, Anhui 230032, P. R. China.

[2]Hefei National Research Center for Physical Sciences at the Microscale, University of Science and Technology of China, Hefei, Anhui 230026, P. R. China.

[3]National Clinical Research Centre for Oral Diseases, Shanghai Key Laboratory of Stomatology and Shanghai Research Institute of Stomatology, Department of Oral Surgery, Shanghai Ninth People's Hospital College of Stomatology, Shanghai Jiao Tong University School of Medicine, Shanghai 200011, China.

[4]Department of Chemistry, Carnegie Mellon University, Pittsburgh, PA, 15213, United States

[*]Corresponding authors: ybsong860@ahmu.edu.cn; rongchao@andrew.cmu.edu; wangmohan15@126.com;

[†]Tingting Xu and Jie Kong contributed equally to this work.

1. Chemicals

Chloroauric acid ($HAuCl_4 \cdot 3H_2O$, ≥ 99.99%), Tetraoctylammonium bromide (TOAB, ≥ 98%), Triphenylphosphine (Ph_3P, ≥ 98.8%), Cuprous bromide (CuBr, 98.0%), Selenophenol (PhSeH, ≥ 99.9%), Sodium borohydride ($NaBH_4$, ≥ 98%), Folic acid-PEG-Amine (NH_2-PEG-FA, 97%, Mw550), Tetraethyl orthosilicate (TEOs, 99.99%), Triethylamine (TEA, ≥ 99.5%), Cetyl trimethyl ammonium bromide (CTAB, 99%), Ammonium hydroxide ($NH_3 \cdot H_2O$), Sodium hydroxide (NaOH, ≥ 98.0%), 9, 10-anthracene bis (methylene) dimalonic acid (ABDA, 99.0%), 3,3′,5,5′-tetramethylbenzidine (TMB, ≥ 99.0%), 5,5-dimethyl-1-pyrroline *N*-oxide (DMPO, 97%), 2,2,6,6-tetramethylpiperidine (TEMP, ≥ 98.0 %), Thiazolyl blue tetrazolium bromide (MTT, > 99.0%), Toluene (≥ 99.9%), Methyl alcohol (MeOH, ≥ 99.9%), Ethanol (EtOH, ≥ 99.9%), Dimethyl sulfoxide (DMSO, ≥ 99.5%), Dichloromethane (DCM, ≥ 99.9%), Deionized (DI) water. All reagents were commercially available and used without further purification.

2. Experimental Methods

Synthesis of Au_{60} NC

The Au_{60} NC were synthesized on the basis of our previous work.[S1] Briefly, 800 μL $HAuCl_4 \cdot 3H_2O$ (157.5 mg, 0.4 mmol) solution and 255.0 mg TOAB were added to 10 mL toluene and vigorously stirred for 10 min. After completing the phase transfer, the clear water phase was removed from the bottom of the flask. Then, the mixed solution was cooled to 0 °C and placed in an ice bath for 30 min without stirring. Subsequently, 310.0 mg Ph_3P was added to the flask under stirring condition. 5 min later, 80.0 mg $NaBH_4$ was dissolved in 5ml ice-cold water and rapidly added in the above solution. After 30 min, 50 μL of PhSeH (4.76 mmol) was added to the flask. The reaction was kept stirring at a low temperature for 24 hours. After the reaction was complete, the aqueous phase was removed from the solution. The organic phase was evaporated by rotary evaporator and washed with ethanol several times to remove the excess regents until pure Au_{60} NC were obtained.

Synthesis of Ph_3P-Cu(I)-Br

89.2 mg CuBr and 10 mL EtOH were added to a round-bottom flask and stirred for 10 min. Then, 104.8 mg Ph_3P was introduced into the mixture and stirred for 2 h. The above solution was centrifuged to separate the supernatant, and the precipitate was washed three times with ethanol. The final product, Ph_3P-Cu(I)-Br complex, was then dried for further use.

Synthesis of SiO_2

12 mL EtOH was added to 24 mL DI water to form a mixture, which was placed in a round-bottom flask. Subsequently, 50 μg CTAB, 300 μL TEA, 300 μL ammonium hydroxide and 300 μL TEOS were successively added to the flask, and the reaction continued for 8 h. The resulting mixture was then centrifugally washed with DI water and EtOH for several times. After drying, CTAB was removed by calcination at 550 ℃ for 5 h. Next, the surface of SiO_2 was etched with NaOH ($m_{NaOH} : m_{SiO2} = 2 : 1$). After 20 min, the mixture was centrifuged and the precipitate washed twice with DI water. Finally, the SiO_2 with porous structure was obtained.

The X-ray diffraction (XRD) pattern reveals that the synthesized SiO_2 corresponds to the standard card (JCPDS: 39-1425), indicating successful preparations of porous SiO_2 through

NaOH etching (Figure S10A). Additionally, the transmission electron microscopy (TEM) diagram demonstrates its a porous irregular spherical structure (Figure S10B).

Synthesis of Cu_xAu_{61-x}@SiO_2

100 mg SiO_2 was dispersed in 16 mL ethanol and 20 mg Cu_xAu_{61-x} NC was dissolved in 100 μL DMSO. Subsequently, the Cu_xAu_{61-x} NC solution is gradually added to the ethanol dispersion of SiO_2 under stirring conditions. After stirring for 4 h, the mixed solution was centrifuged at 8000 rpm. As shown in Figure S9, the supernatant shows colorless and no characteristic absorption peaks of Cu_xAu_{61-x} NC, indicating the Cu_xAu_{61-x} NCs were loaded onto the SiO_2. Finally, the black precipitation was collected, which is the Cu_xAu_{61-x}@SiO_2.

Synthesis of Cu_xAu_{61-x}@SiO_2–FA

The assembly of the targeted groups was referred to the previously reported literature.[S2] 2 mg/mL of Cu_xAu_{61-x}@SiO_2 was mixed with 2 mg/mL of NH_2–PEG–FA solution under ultrasound for 5 min. Subsequently, the pH of mixed solution was adjusted to 7.4 by NaOH and stirred for 4 h. After the reaction is complete, the precipitation was collected by centrifugation and washed three times before remaining for further use. All the concentrations used in this work were calculated only based on the amount of Cu_xAu_{61-x}. For example, 200 μg/mL: dispersing 12 mg of Cu_xAu_{61-x}@SiO_2 (corresponding to 2 mg Cu_xAu_{61-x}) into 10 mL water.

Characterization

One piece crystal of Cu_xAu_{61-x} NC with high quality was selected and performed on a Bruker D8 Venture X-ray single crystal diffractometer. Ultraviolet visible (UV-vis) absorption spectra were mearsured by a UV-6000PC spectrophotometer. Infrared imaging devices (FLIR E85, FLIR Systems) were used to record temperature changes of solution. Electron spin resonance spectrometer (ESR, EMXnano) was utilized to capture free radicals. Transmission electron microscopy (TEM, FEI Tecnai F20) displayed the morphology of the sample. Fluorescence images were filmed by confocal laser scanning microscopy (CLSM, LMS-800). Cell viability was measured by microplate reader (SpectraMax iD3). Matrix assisted laser mass spectrometer (MALID, iDplus Performance) were from Shimadzu Instrument Co., LTD. All the emission spectra were detected using a fluorescence spectrometer (Omnifluo990LSO). Luminescence imaging photographs were taken using a full-spectrum fluorescence *in vivo* imaging system (Monet IGS-1500).

The femtosecond transient absorption spectroscopy measurements were performed on a home-built pump−probe set-up. The laser pulse (800 nm, 35 fs pulse width, 1 kHz repetition rate) was generated by a regeneratively amplified Ti: sapphire laser (Coherent Astrella-Tunalbe-USP, USA). The output of the pulse is divided into two beams with a beam splitter. The 400-pump beam was produced by doubling the 800 nm pulse with a beta barium borate crystal (type I, 0.5 mm thickness); the power of pump pulse was set as 50 μw. The probe pulse was delayed by a computer-controlled optical delay line and then focused on a thin sappire plate to generate the white light supercontinuum which split into two beams by using a broadband 50/50 beam splitter as the signal and reference beams (440 - 790 nm). The focused pump and probe pulses overlapped into a sample cuvette. The mutual polarization between the pump and probe pulses was set to the 54.7° by placing a half-wave plate in the pump beam.

There is no photodegrading after fs-TA experiments by checking the steady-state absorption spectra.

The ns-TA spectra were measured by a commercial spectrometer (Time-Tech Spectra). The generation of the pump beam is the same as that in fs-TA. The probe beam was generated from a supercontinuum laser (LEUKOS-DISCO, French) with the spectral region from 410 to 950 nm, the repetition rate is 2 kHz, pulse width is 700 ps - 1 ns. There is also no photodegrading after ns-TA experiments by checking the steady-state absorption spectra.

Table S1. Crystal data and structure refinement for Cu_xAu_{61-x}.

Empirical formula	$C_{270}H_{225}Au_{55.73}Br_2Cu_{5.28}P_{10}Se_{17}$
CCDC	2432419
Formula weight	16592.46
Temperature/K	170 (2)
Crystal system	monoclinic
Space group	$P2_1/c$
a/Å	32.147(5)
b/Å	27.313(5)
c/Å	46.355(8)
α/°	90
β/°	107.496(10)
γ/°	90
Volume/Å^3	38818(12)
Z	4
ρ_{calc}g/cm^3	2.839
μ/mm^{-1}	41.446
F(000)	28793.0
Crystal size/mm^3	0.82 × 0.45 × 0.31
Radiation	CuK_α (λ = 1.54184)
2θ range for data collection/°	4.166 to 136.962
Index ranges	$-38 \leq h \leq 33, -28 \leq k \leq 32, -54 \leq l \leq 55$
Reflections collected	312947
Independent reflections	70302 [R_{int} = 0.1040, R_{sigma} = 0.0691]
Data/restraints/parameters	70302/5087/2768
Goodness-of-fit on F^2	0.997
Final R indexes [I>=2σ (I)]	R_1 = 0.0930, wR_2 = 0.2509
Final R indexes [all data]	R_1 = 0.1199, wR_2 = 0.2760
Largest diff. peak/hole / e Å^{-3}	4.80/–5.78

Table S2. The Au/Cu occupancies in the Cu_xAu_{61-x} NC (avg x = 5.28).

site	1	2	3	4	5	6	7	8	9	10	11
Au(%)	71.1	50.5	56.1	45.4	30.6	81.8	53.7	30.8	85.5	66.6	0
Cu(%)	28.9	49.5	43.9	54.6	69.4	18.2	46.3	69.2	14.5	33.4	100

Supporting figures:

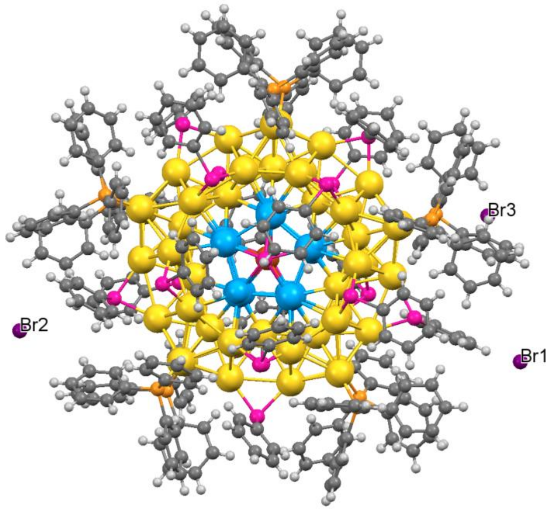

Figure S1. Total structure of $[Cu_xAu_{61-x}Se_2(PhSe)_{15}(Ph_3P)_{10}]Br_2$ ($1 \leq x \leq 11$). Color code: yellow = Au; blue = Au/Cu; brown = Cu; magenta = Se; orange = P, purple = Br, gray = C; white = H. Note: The occupancy of Br1, Br2 and Br3 is 1, 0.5 and 0.5, respectively.

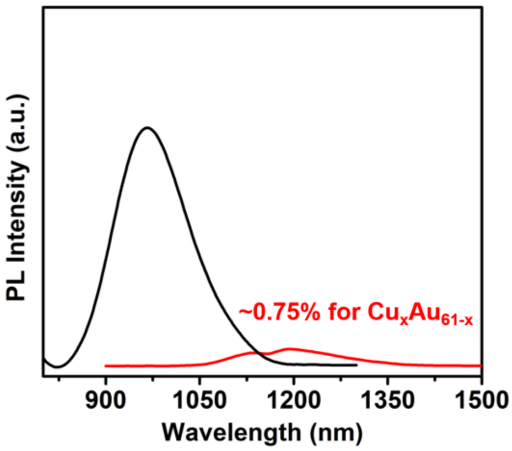

Figure S2. The relative quantum yield of the Cu_xAu_{61-x} NC (PL: red curve) determined by comparing to the rod-like $[Au_{25}(SC_2H_4Ph)_5(PPh_3)_{10}X_2]^{2+}$ (X = Cl/Br) NC (PL: black curve) in dichloromethane (λ_{ex} = 410 nm). The integrated PL peak areas were used in the determination.

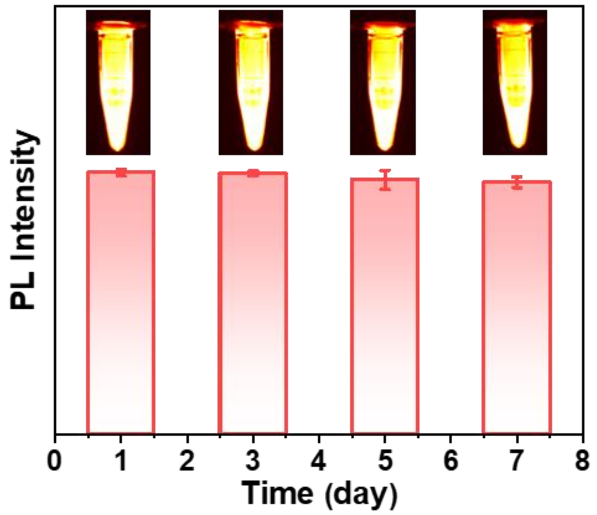

Figure S3. Photostability of Cu_xAu_{61-x} for 7 days in DMSO, inset: the corresponding NIR-II images.

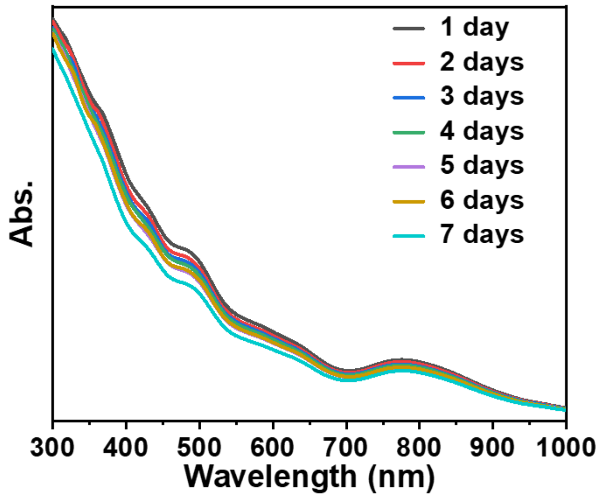

Figure S4. Stability of optical characteristic absorption peaks of Cu_xAu_{61-x} NC of 7 days in DMSO.

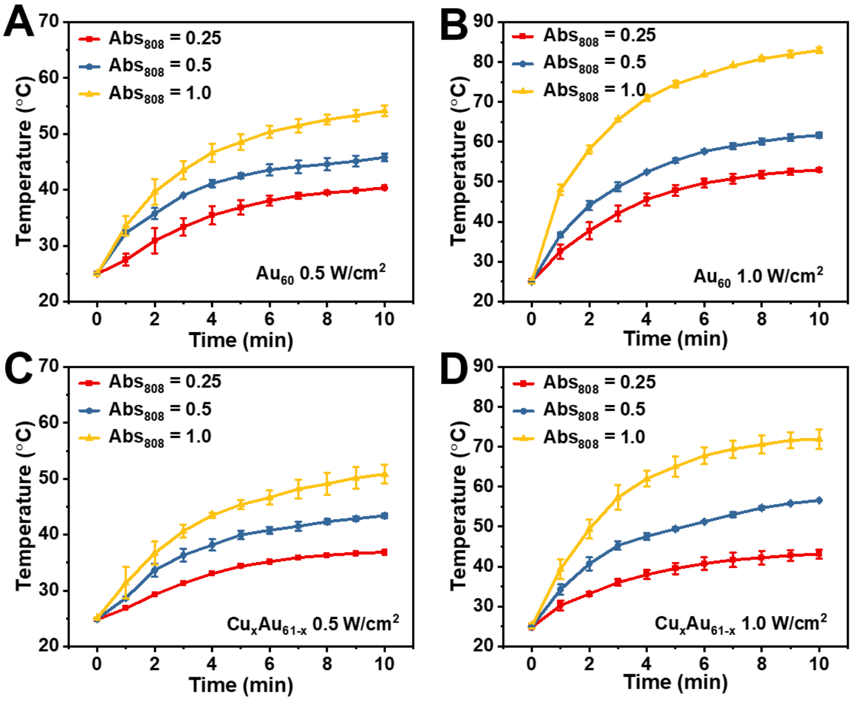

Figure S5. Temperature changes of Au_{60} and Cu_xAu_{61-x} NCs in DMSO with different absorbances (0.25, 0.5 and 1.0) upon irradiation at 808 nm: (A) Au_{60} NC at 0.5 W/cm^2, (B) Au_{60} NC at 1.0 W/cm^2, (C) Cu_xAu_{61-x} NC at 0.5 W/cm^2, (D) Cu_xAu_{61-x} NC at 1.0 W/cm^2.

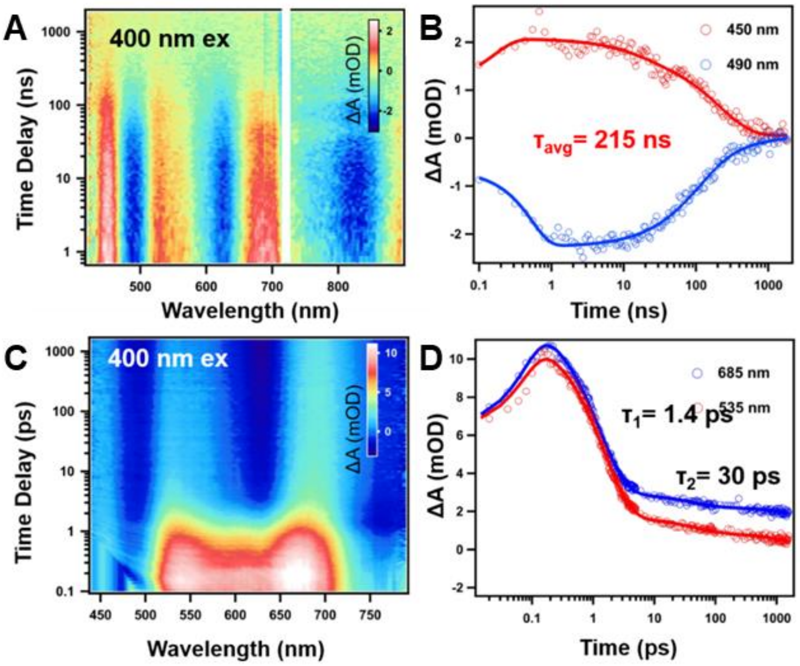

Figure S6. (A) ns-TA data map of Cu_xAu_{61-x} NC in DCM under 400 nm excitation. (B) ns-TA kinetic traces and corresponding fits at selected probe wavelengths. (C) fs-TA data map of Cu_xAu_{61-x} NC in DCM under 400 nm excitation. (D) fs-TA kinetic traces and corresponding fits at selected probe wavelengths.

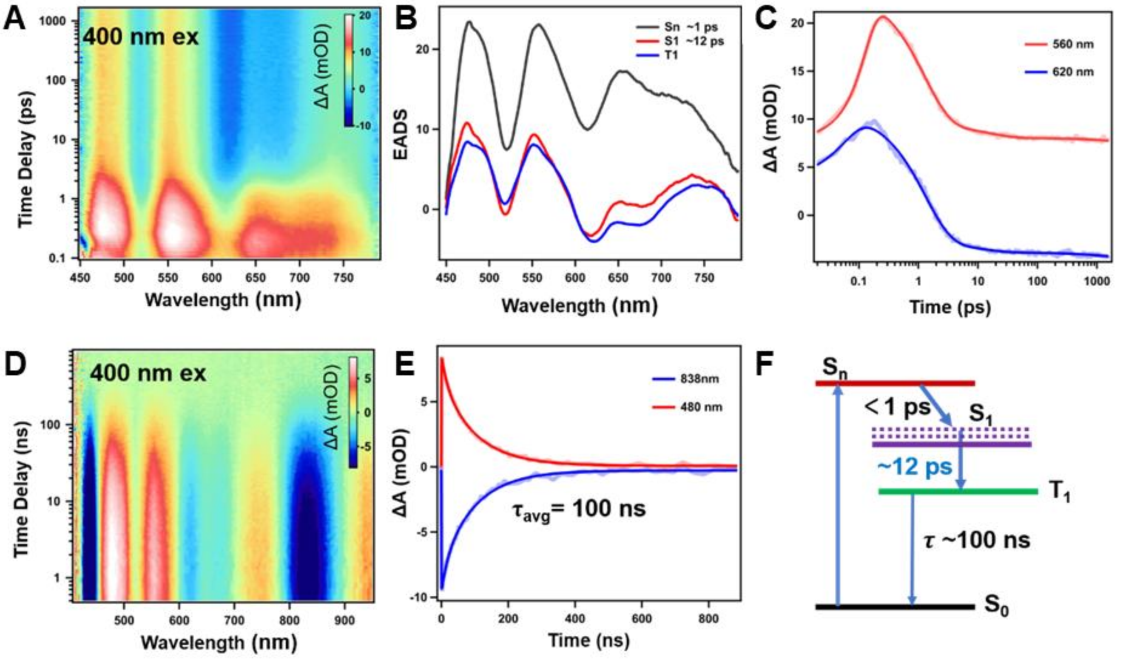

Figure S7. (A) fs-TA data map of Au_{60} in DMSO with 400 nm excitation. (B) Global analysis results derived from fs-TA of Au_{60} NC. (C) fs-TA kinetic traces and corresponding fits at selected probe wavelengths. (D) ns-TA data map of Au_{60} in DMSO with 400 nm excitation. (E) ns-TA kinetic traces and corresponding fits at selected probe wavelengths. (F) The diagrams of excited-state deactivation mechanism of Au_{60} NC.

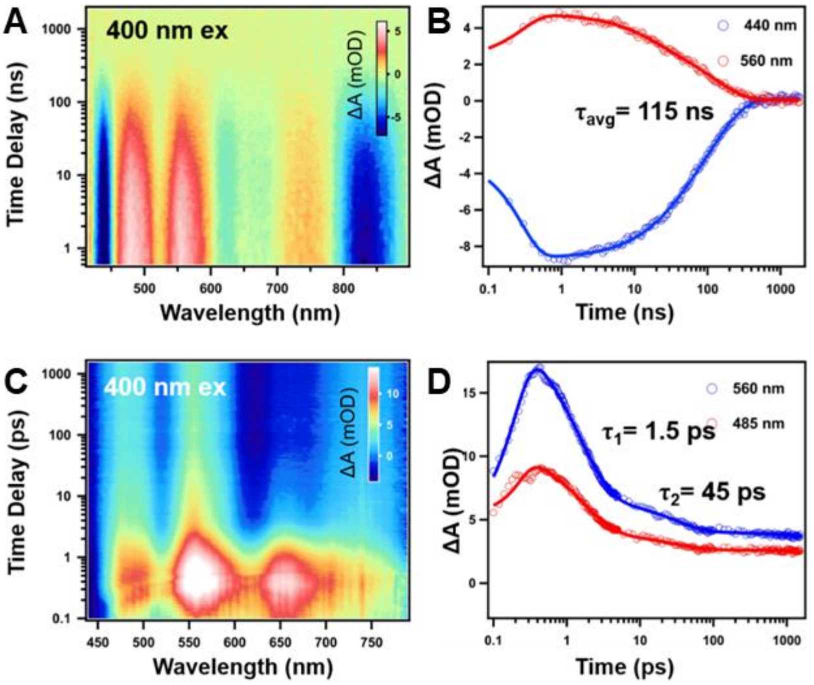

Figure S8. (A) ns-TA data map of Au_{60} in DCM with 400 nm excitation. (B) ns-TA kinetic traces and corresponding fits at selected probe wavelengths. (C) fs-TA data map of Au_{60} in DCM with 400 nm excitation. (D) fs-TA kinetic traces and fits at selected probe wavelengths.

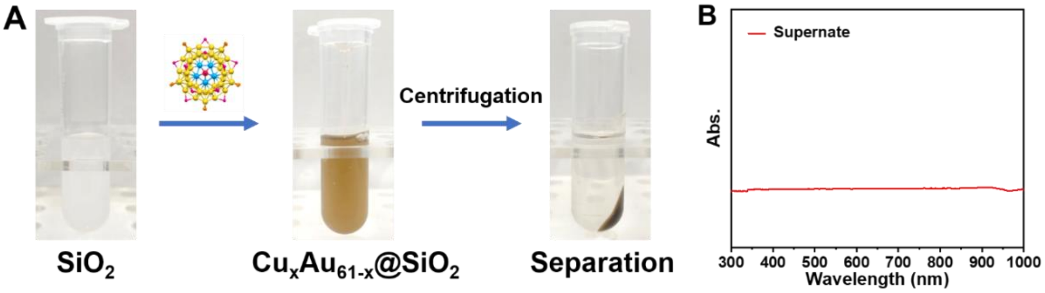

Figure S9. (A) The process for depositing Cu_xAu_{61-x} NCs onto SiO_2. (B) UV-vis spectrum of the supernatant after centrifugation.

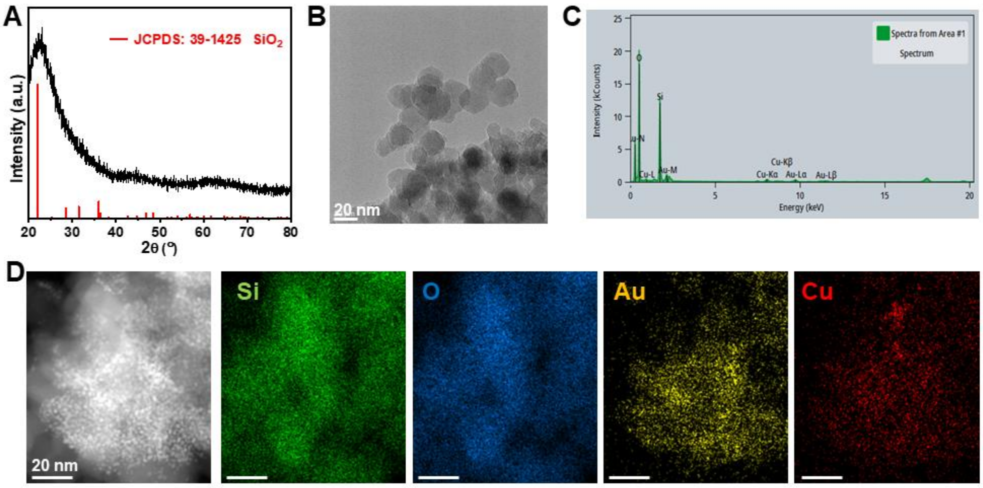

Figure S10. (A) Powder XRD pattern and (B) TEM image of SiO_2, (C) EDS spectrum and (D) Representative STEM image and elemental mapping images of Si, O, Au and Cu of $Cu_xAu_{61-x}@SiO_2$.

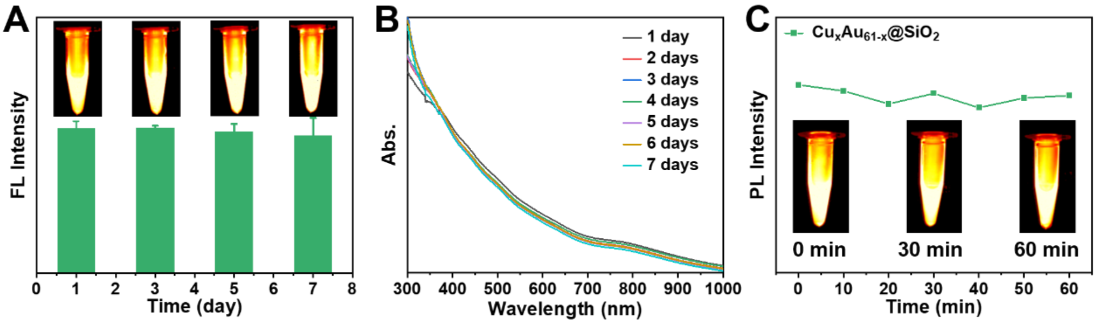

Figure S11. (A) Photostability of $Cu_xAu_{61-x}@SiO_2$ for 7 days in water, inset: the corresponding NIR-II images. (B) Stability of optical characteristic absorption peaks of $Cu_xAu_{61-x}@SiO_2$ for 7 days in water. (C) Photostability of $Cu_xAu_{61-x}@SiO_2$ excited with an 808 nm laser for 60 min, inset: the corresponding NIR-II images.

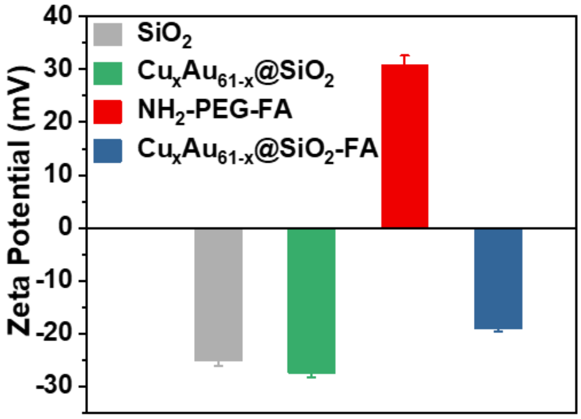

Figure S12. Zeta potential of SiO_2, $Cu_xAu_{61-x}@SiO_2$, NH_2-PEG-FA and $Cu_xAu_{61-x}@SiO_2$-FA.

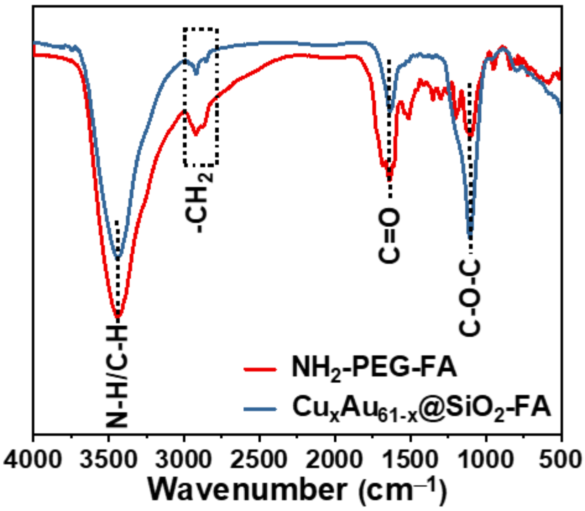

Figure S13. FT-IR spectra of $Cu_xAu_{61-x}@SiO_2$-FA and NH_2-SiO_2-FA.

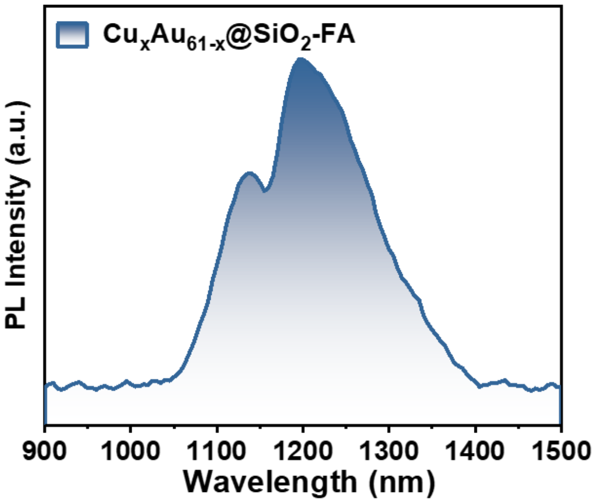

Figure S14. NIR-II fluorescence spectrum of $Cu_xAu_{61-x}@SiO_2$-FA.

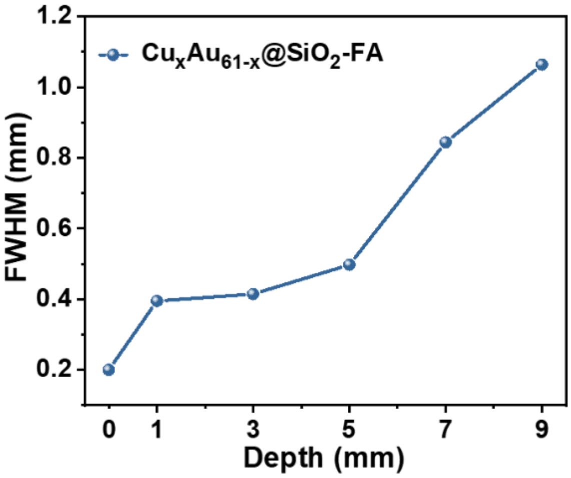

Figure S15. The full width at half-maximum of $Cu_xAu_{61-x}@SiO_2$-FA at different penetration depths.

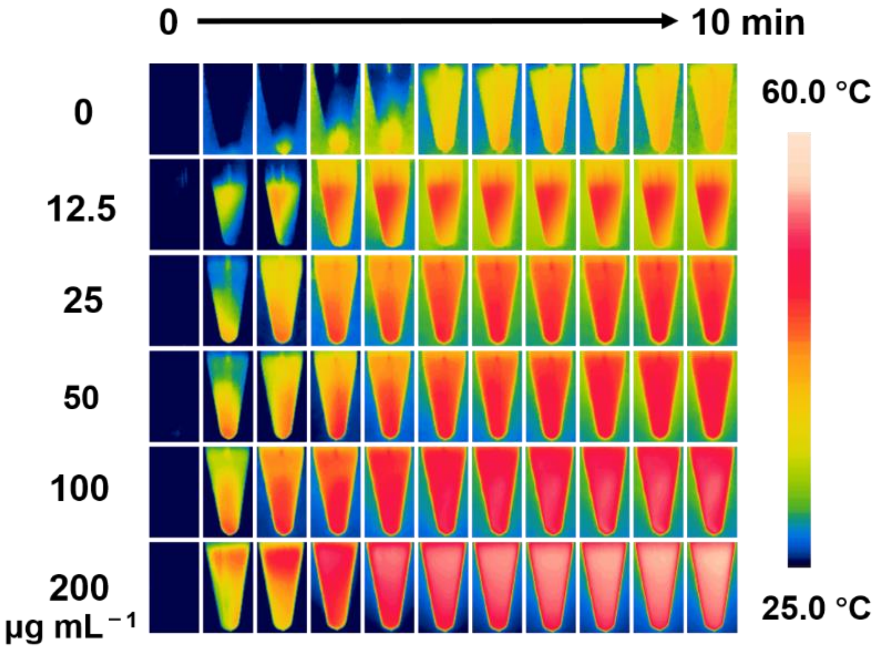

Figure S16. Infrared thermography image of Cu_xAu_{61-x}@SiO_2–FA at different concentrations upon irradiation at 808 nm (1.0 W/cm^2).

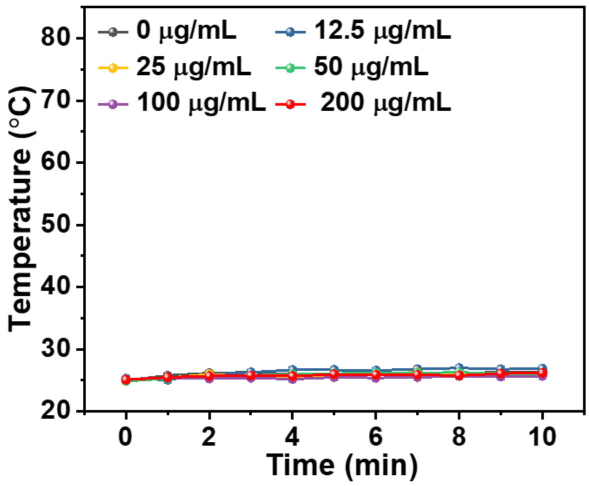

Figure S17. Temperature variation curve of SiO_2-FA nanoparticle solution with different concentrations under 808 nm laser irradiation with a power density of 1.0 W/cm^2.

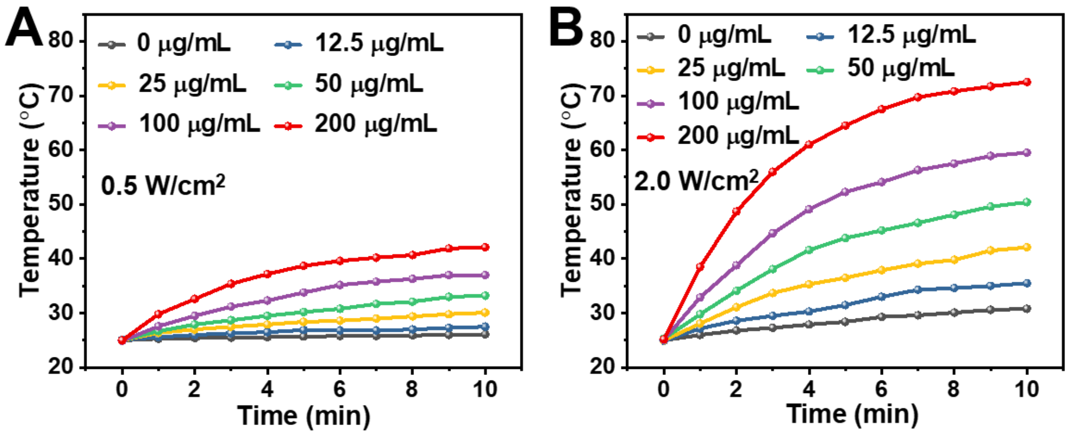

Figure S18. Temperature change of Cu_xAu_{61-x}@SiO_2–FA dispersions at different concentrations upon irradiation at 808 nm for 10 min: (A) 0.5 W/cm^2, (B) 2.0 W/cm^2.

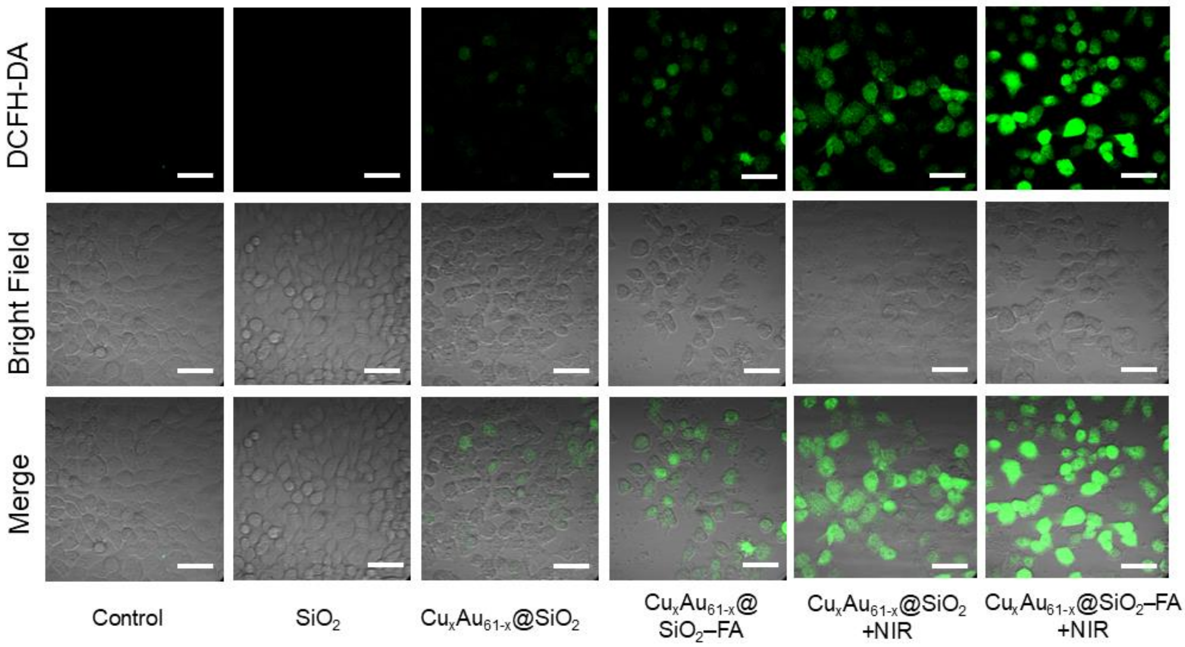

Figure S19. Confocal laser scanning microscopy (CLSM) observation using DCFH-DA staining, after 4 h of incubation with different treatments (Scale bar = 50 μm)

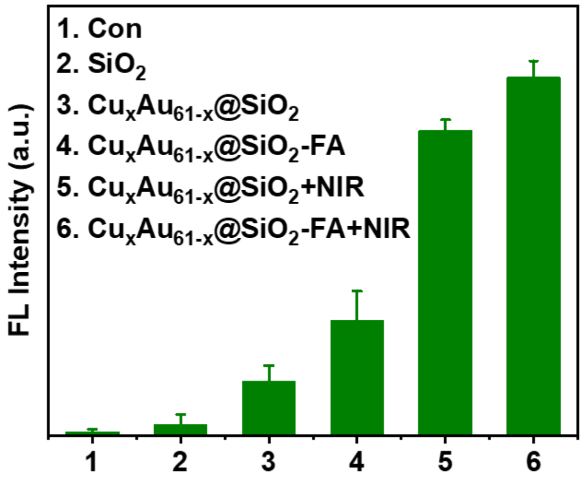

Figure S20. Quantitative analysis of confocal laser scanning microscopy (CLSM) observation using DCFH-DA staining.

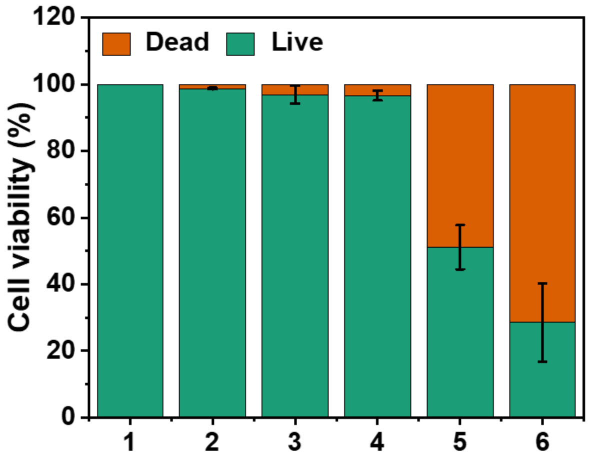

Figure S21. Quantitative analysis of Fluorescend microscopy images of CAL27 cells after different treatments (1: Con, 2: SiO_2, 3: Cu_xAu_{61-x}@SiO_2, 4: Cu_xAu_{61-x}@SiO_2-FA, 5: Cu_xAu_{61-x}@SiO_2+NIR. 6: Cu_xAu_{61-x}@SiO_2–FA+NIR).

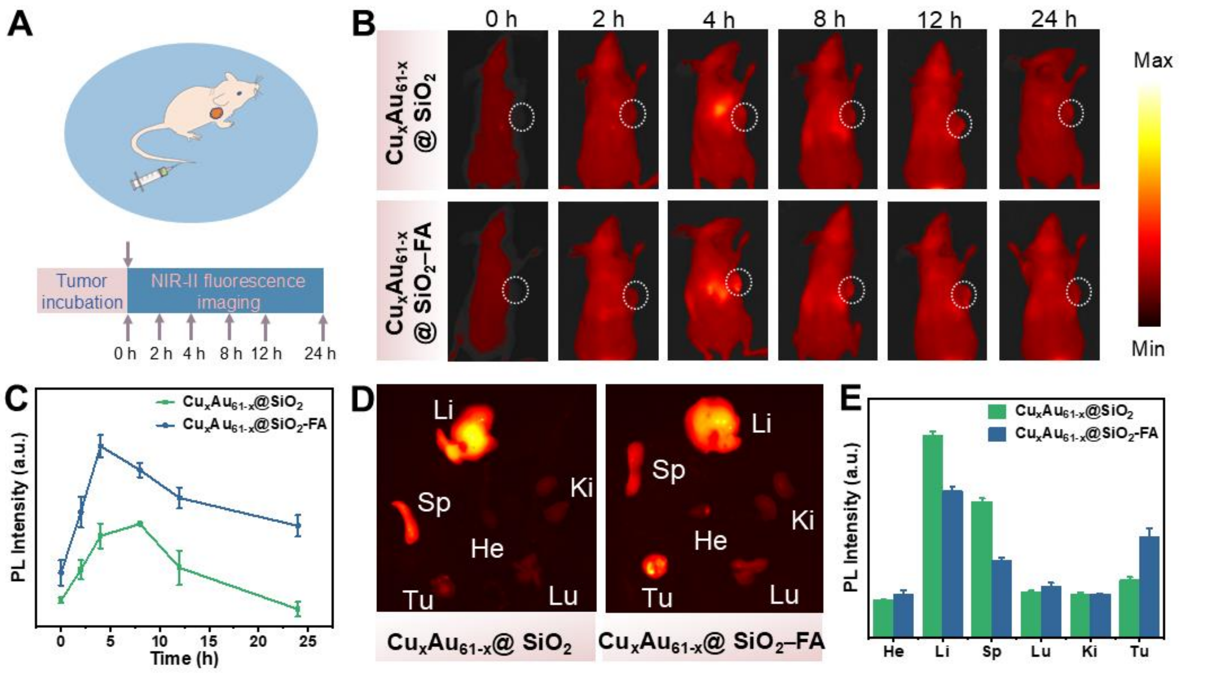

Figure S22. (A) Schematic illustration of the *in vivo* biodistribution experiment of $Cu_xAu_{61-x}@SiO_2$–FA under 808 nm irradiation. (B) NIR-II PL imaging in BALB/c nude mice at different time points following intravenous injection of $Cu_xAu_{61-x}@SiO_2$–FA, and (C) corresponding PL intensity curves of tumors over time ($n = 3$). (D) NIR-II PL imaging of major organs and tumors at 24 h post-injection (He: heart, Li: Liver, Sp: Spleen, Lu: lung, Ki: kidney, Tu: tumor) ($n = 3$). (E) Semi-quantitative biodistribution analysis based on PL intensity in tumors and major organs ($n = 3$).

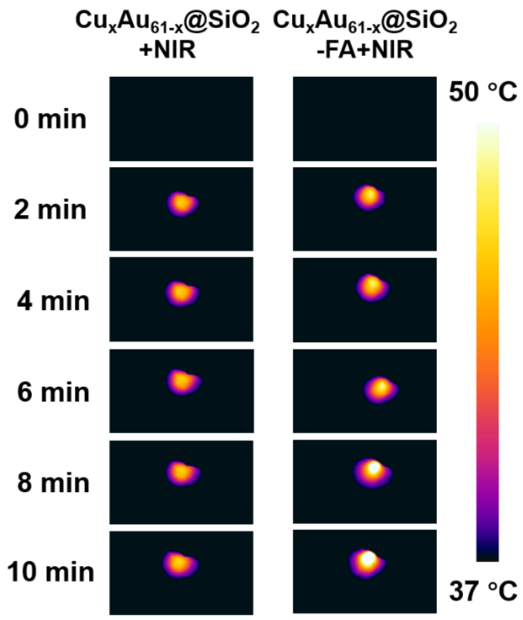

Figure S23. Infrared thermal images of mice post-injection of $Cu_xAu_{61-x}@SiO_2$ and $Cu_xAu_{61-x}@SiO_2$–FA under different irradiation durations with the temperature window of 37-50 ºC.

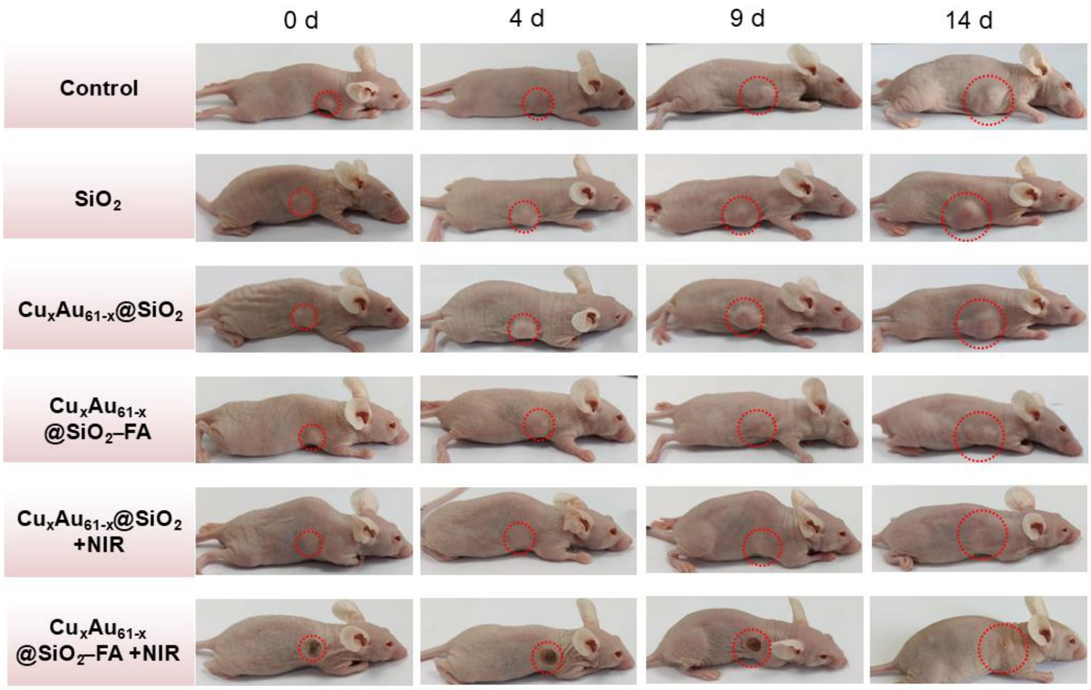

Figure S24. Photos of mice in different treatment groups.

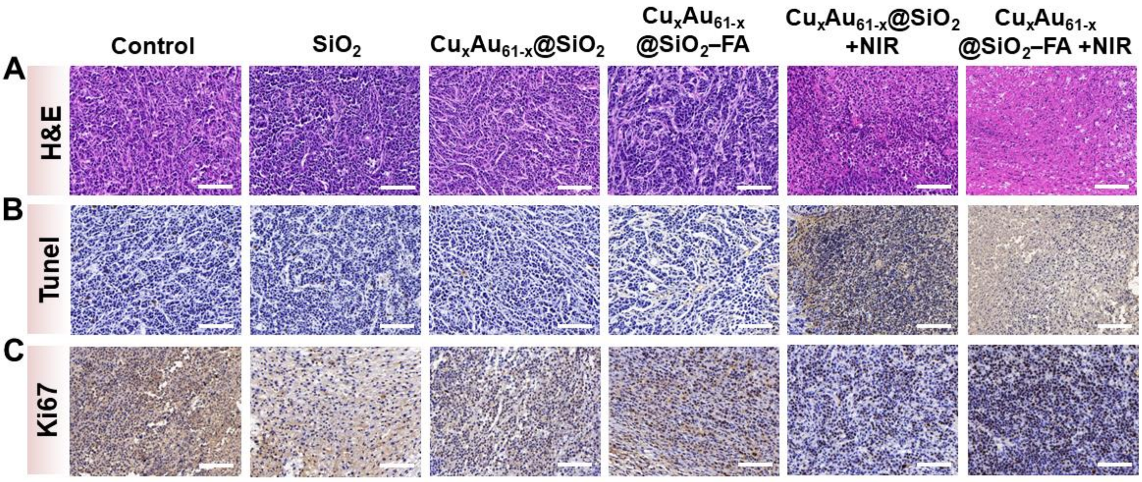

Figure S25. (A) H&E, (B) TUNEL and (C) Ki67 staining images of the excised tumors after different treatment groups on the 15th day (scale bar: 100 μm)

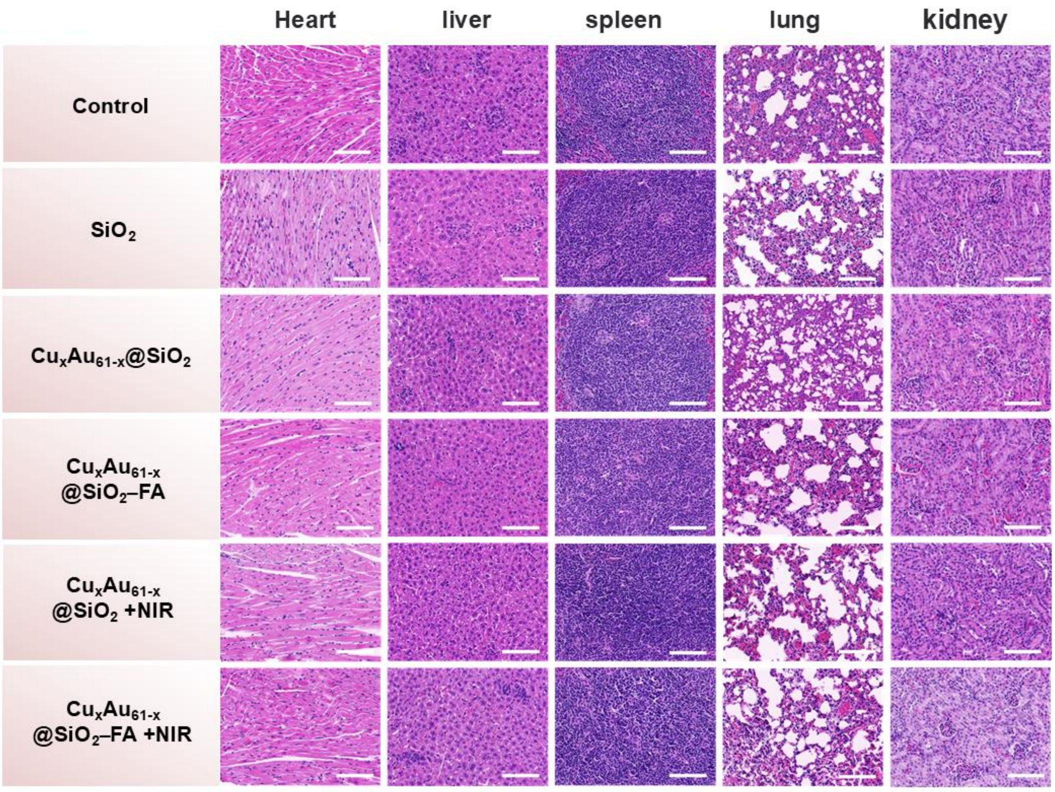

Figure S26. H&E staining images of major organs (heart, liver, spleen, lung and kidney) under different treatments (scale bar: 100 μm).

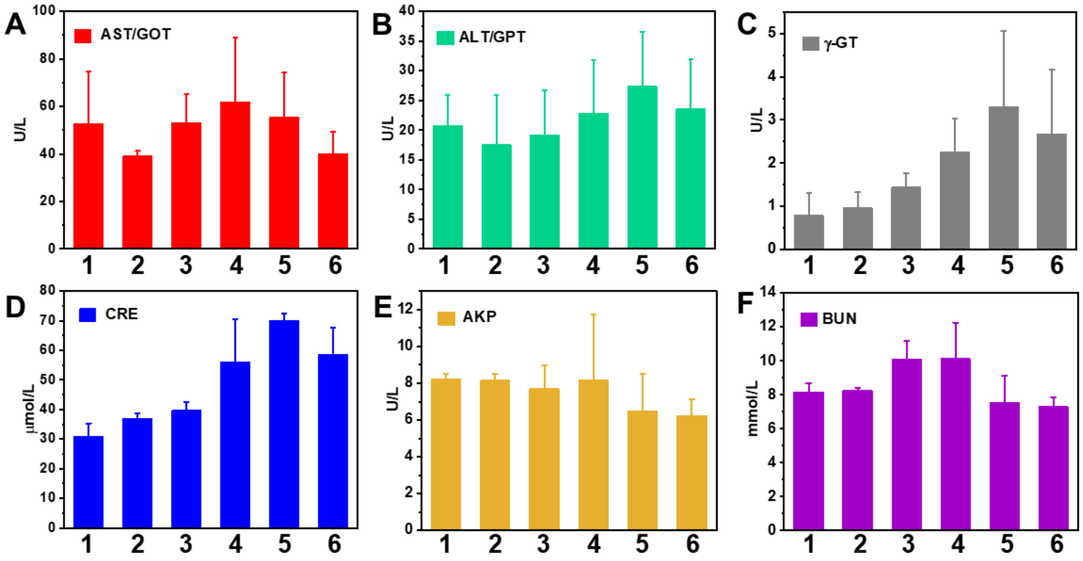

Figure S27. Biochemical analysis of mouse blood after injecting Cu_xAu_{61-x}@SiO_2-FA through tail vein (*n* = 3), (a) glutamic oxalacetic transaminase (AST/GOT), (b) glutamic-pyruvic transaminas (ALT/GPT), (c) γ-Glutamyl transaminase (γ-GT), (d) creatinine (CRE), (e) alkaline phosphatase (AKP), (f) blood urea nitrogen (BUN), 1: Control, 2: SiO_2, 3: Cu_xAu_{61-x}@SiO_2, 4: Cu_xAu_{61-x}@SiO_2–FA, 5: Cu_xAu_{61-x}@SiO_2 +NIR, 6: Cu_xAu_{61-x}@SiO_2–FA +NIR.

Supporting references

[S1] Y. Song, F. Fu, J. Zhang, J. Chai, X. Kang, P. Li, S. Li, H. Zhou, M. Zhu, The Magic Au_{60} Nanocluster: A New Cluster-Assembled Material with Five Au_{13} Building Blocks. *Angew. Chem. Int. Ed.* **2015**, *127*, 8550-8554.

[S2] X. Ren, W. Liu, H. Zhou, J. Wei, C. Mu, Y. Wan, X. Yang, A. Nie, Z. Liu, X. Yang, Z. Luo, Biodegradable 2D GeP Nanosheets with High Photothermal Conversion Efficiency for Multimodal Cancer Theranostics. *Chem. Eng. J.* **2022**, *431*, 134176.
