# Supplementary material for: Triple‐Functional CuxAu61‐x Nanoclusters with NIR‐II Photoluminescence, Photothermal and Photodynamic Properties and Their Bio‐Application
Source: Adv Sci (Weinh). 2025 Jul 18;12(39):e09283. doi: 10.1002/advs.202509283 (PMC12533204; doi:10.1002/advs.202509283)

checkCIF/PLATON report

You have not supplied any structure factors. As a result the full set of tests cannot be run.

THIS REPORT IS FOR GUIDANCE ONLY. IF USED AS PART OF A REVIEW PROCEDURE FOR PUBLICATION, IT SHOULD NOT REPLACE THE EXPERTISE OF AN EXPERIENCED CRYSTALLOGRAPHIC REFEREE.

No syntax errors found. CIF dictionary Interpreting this report

Datablock: cu_20240720_ys_aucu60_0m

```
Bond precision:   C-C = 0.0416 A          Wavelength=1.54184

Cell:             a=32.147(5)     b=27.313(5)          c=46.355(8)
                  alpha=90        beta=107.496(10)     gamma=90
Temperature:      170 K
```

	Calculated	Reported
Volume	38818(12)	38818(12)
Space group	P 21/c	P 1 21/c 1
Hall group	-P 2ybc	-P 2ybc
Moiety formula	C270 H225 Au55.72 Cu5.28 P10 Se17, 2(Br) [+ solvent]	C270 H225 Au55.721 Cu5.279 P10 Se17, 2(Br)
Sum formula	C270 H225 Au55.72 Br2 Cu5.28 P10 Se17 [+ solvent]	C270 H225 Au55.72 Br2 Cu5.28 P10 Se17
Mr	16592.06	16592.46
Dx,g cm-3	2.839	2.839
Z	4	4
Mu (mm-1)	41.444	41.446
F000	28792.2	28793.0
F000'	27781.41	
h,k,lmax	38,32,55	38,32,55
Nref	71434	70302
Tmin,Tmax	0.000,0.000	0.245,0.753
Tmin'	0.000	

```
Correction method= # Reported T Limits: Tmin=0.245 Tmax=0.753
AbsCorr = MULTI-SCAN

Data completeness= 0.984          Theta(max)= 68.481
```

```
R(reflections)= 0.0930( 44554)                 wR2(reflections)=
                                               0.2760( 70302)
S = 0.997                Npar= 2768
```

The following ALERTS were generated. Each ALERT has the format
test-name_ALERT_alert-type_alert-level.
Click on the hyperlinks for more details of the test.

Alert level B

```
PLAT196_ALERT_1_B No TEMP record and measurement temperature .NE.         293 Degree
PLAT241_ALERT_2_B High   'MainMol' Ueq as Compared to Neighbors of       C203 Check
PLAT242_ALERT_2_B Low    'MainMol' Ueq as Compared to Neighbors of         P9 Check
PLAT242_ALERT_2_B Low    'MainMol' Ueq as Compared to Neighbors of        P10 Check
PLAT332_ALERT_2_B Large Phenyl C-C Range     C1        -C154     .        0.29 Ang.
PLAT342_ALERT_3_B Low Bond Precision on  C-C Bonds ...............    0.04159 Ang.
PLAT410_ALERT_2_B Short Intra H...H Contact  H185      ..H205     .       1.82 Ang.
                                                      x,y,z  =      1_555 Check
```

Alert level C

```
PLAT084_ALERT_3_C High wR2 Value (i.e. > 0.25) ...................       0.28 Report
PLAT218_ALERT_3_C Constrained U(i,j) Components(s) for C33                   6 Check
PLAT218_ALERT_3_C Constrained U(i,j) Components(s) for C87                   6 Check
PLAT218_ALERT_3_C Constrained U(i,j) Components(s) for C93                   6 Check
PLAT218_ALERT_3_C Constrained U(i,j) Components(s) for C107                  6 Check
PLAT218_ALERT_3_C Constrained U(i,j) Components(s) for C123                  6 Check
PLAT218_ALERT_3_C Constrained U(i,j) Components(s) for C129                  6 Check
PLAT220_ALERT_2_C NonSolvent   Resd 1  C   Ueq(max)/Ueq(min) Range         4.5 Ratio
PLAT222_ALERT_3_C NonSolvent Resd 1  H   Uiso(max)/Uiso(min) Range         4.4 Ratio
PLAT241_ALERT_2_C High   'MainMol' Ueq as Compared to Neighbors of        C15 Check
PLAT241_ALERT_2_C High   'MainMol' Ueq as Compared to Neighbors of        C32 Check
PLAT241_ALERT_2_C High   'MainMol' Ueq as Compared to Neighbors of        C45 Check
PLAT241_ALERT_2_C High   'MainMol' Ueq as Compared to Neighbors of        C63 Check
PLAT241_ALERT_2_C High   'MainMol' Ueq as Compared to Neighbors of        C84 Check
PLAT241_ALERT_2_C High   'MainMol' Ueq as Compared to Neighbors of        C93 Check
PLAT241_ALERT_2_C High   'MainMol' Ueq as Compared to Neighbors of       C117 Check
PLAT241_ALERT_2_C High   'MainMol' Ueq as Compared to Neighbors of       C172 Check
PLAT241_ALERT_2_C High   'MainMol' Ueq as Compared to Neighbors of       C190 Check
PLAT241_ALERT_2_C High   'MainMol' Ueq as Compared to Neighbors of       C202 Check
PLAT241_ALERT_2_C High   'MainMol' Ueq as Compared to Neighbors of       C214 Check
PLAT241_ALERT_2_C High   'MainMol' Ueq as Compared to Neighbors of       C233 Check
PLAT241_ALERT_2_C High   'MainMol' Ueq as Compared to Neighbors of       C246 Check
PLAT241_ALERT_2_C High   'MainMol' Ueq as Compared to Neighbors of       C263 Check
PLAT242_ALERT_2_C Low    'MainMol' Ueq as Compared to Neighbors of        Se3 Check
PLAT242_ALERT_2_C Low    'MainMol' Ueq as Compared to Neighbors of        Se7 Check
PLAT242_ALERT_2_C Low    'MainMol' Ueq as Compared to Neighbors of        Se9 Check
PLAT242_ALERT_2_C Low    'MainMol' Ueq as Compared to Neighbors of       Se13 Check
PLAT242_ALERT_2_C Low    'MainMol' Ueq as Compared to Neighbors of       Se16 Check
PLAT242_ALERT_2_C Low    'MainMol' Ueq as Compared to Neighbors of         P2 Check
PLAT242_ALERT_2_C Low    'MainMol' Ueq as Compared to Neighbors of         P3 Check
PLAT242_ALERT_2_C Low    'MainMol' Ueq as Compared to Neighbors of         P4 Check
PLAT242_ALERT_2_C Low    'MainMol' Ueq as Compared to Neighbors of         P5 Check
PLAT242_ALERT_2_C Low    'MainMol' Ueq as Compared to Neighbors of         P6 Check
PLAT242_ALERT_2_C Low    'MainMol' Ueq as Compared to Neighbors of         P7 Check
```

```
PLAT242_ALERT_2_C Low    'MainMol' Ueq as Compared to Neighbors of        P8 Check
PLAT260_ALERT_2_C Large Average Ueq of Residue Including      Au1      0.110 Check
PLAT260_ALERT_2_C Large Average Ueq of Residue Including      Br1      0.131 Check
PLAT260_ALERT_2_C Large Average Ueq of Residue Including      Br2      0.123 Check
PLAT260_ALERT_2_C Large Average Ueq of Residue Including      Br3      0.177 Check
PLAT331_ALERT_2_C Small Aver Phenyl C-C Dist C28      --C213     .       1.36 Ang.
PLAT332_ALERT_2_C Large Phenyl C-C Range     C25       -C221     .       0.22 Ang.
PLAT369_ALERT_2_C Long   C(sp2)-C(sp2) Bond  C55      - C154     .       1.53 Ang.
PLAT410_ALERT_2_C Short Intra H...H Contact  H119     ..H127     .       1.92 Ang.
                                                      x,y,z  =      1_555 Check
```

Alert level G

```
PLAT002_ALERT_2_G Number of Distance or Angle Restraints on AtSite        26 Note
PLAT003_ALERT_2_G Number of Uiso or U(i,j)  Restrained non-H-Atoms       281 Report
PLAT042_ALERT_1_G Calc. and Reported MoietyFormula Strings  Differ     Please Check
              Calc: C270 H225 Au55.72 Cu5.28 P10 Se17, 2(Br)
              Rep.: C270 H225 Au55.721 Cu5.279 P10 Se17, 2(Br)
PLAT063_ALERT_4_G Crystal Size Possibly too Large for Beam Size ..       0.80 mm
PLAT072_ALERT_2_G SHELXL First  Parameter in WGHT  Unusually Large       0.19 Report
PLAT171_ALERT_4_G The CIF-Embedded .res File Contains EADP Records          8 Report
PLAT172_ALERT_4_G The CIF-Embedded .res File Contains DFIX Records          8 Report
PLAT174_ALERT_4_G The CIF-Embedded .res File Contains FLAT Records          3 Report
PLAT176_ALERT_4_G The CIF-Embedded .res File Contains SADI Records          6 Report
PLAT178_ALERT_4_G The CIF-Embedded .res File Contains SIMU Records          1 Report
PLAT186_ALERT_4_G The CIF-Embedded .res File Contains ISOR Records          1 Report
PLAT187_ALERT_4_G The CIF-Embedded .res File Contains RIGU Records          1 Report
PLAT188_ALERT_3_G A Non-default SIMU Restraint Value has been used     0.0100 Report
PLAT190_ALERT_3_G A Non-default RIGU Restraint Value for First Par     0.0100 Report
PLAT190_ALERT_3_G A Non-default RIGU Restraint Value for SecondPar     0.0100 Report
PLAT191_ALERT_3_G A Non-default SADI Restraint Value has been used     0.0100 Report
PLAT191_ALERT_3_G A Non-default SADI Restraint Value has been used     0.0100 Report
PLAT191_ALERT_3_G A Non-default SADI Restraint Value has been used     0.0100 Report
PLAT191_ALERT_3_G A Non-default SADI Restraint Value has been used     0.0100 Report
PLAT191_ALERT_3_G A Non-default SADI Restraint Value has been used     0.0100 Report
PLAT191_ALERT_3_G A Non-default SADI Restraint Value has been used     0.0100 Report
PLAT299_ALERT_4_G Atom Site Occupancy Constrained at ............         0.5 Check
              Br2     Br3
PLAT301_ALERT_3_G Main Residue  Disorder .............(Resd    1)         3% Note
PLAT302_ALERT_4_G Anion/Solvent/Minor-Residue Disorder (Resd    3)       100% Note
PLAT302_ALERT_4_G Anion/Solvent/Minor-Residue Disorder (Resd    4)       100% Note
PLAT304_ALERT_4_G Non-Integer Number of Atoms in ..... (Resd    3)       0.50 Check
PLAT304_ALERT_4_G Non-Integer Number of Atoms in ..... (Resd    4)       0.50 Check
PLAT606_ALERT_4_G Solvent Accessible VOID(S) in Structure ........          ! Info
PLAT790_ALERT_4_G Centre of Gravity not Within Unit Cell: Resd.  #          3 Note
              Br
PLAT790_ALERT_4_G Centre of Gravity not Within Unit Cell: Resd.  #          4 Note
              Br
PLAT860_ALERT_3_G Number of Least-Squares Restraints ............        5087 Note
PLAT869_ALERT_4_G ALERTS Related to the Use of SQUEEZE  Suppressed          ! Info
PLAT933_ALERT_2_G Number of HKL-OMIT Records in Embedded .res File         19 Note
               -4  1  9,   0  1  3,  -3  1  8,  -3  2  1,  -1  1  1,   3  2  4,
                1  4  0,   3  4  5,  -4  0 10,   6  3  1,   0  1  7,   1  7  5,
               -4  3  3,   0  4  3,  -2  1  6,   2  1  2,   5  0  2,   0  2  8,
                4  3  3,
PLAT941_ALERT_3_G Average HKL Measurement Multiplicity ..........         4.5 Low
```

```
 0 ALERT level A = Most likely a serious problem - resolve or explain
 7 ALERT level B = A potentially serious problem, consider carefully
43 ALERT level C = Check. Ensure it is not caused by an omission or oversight
34 ALERT level G = General information/check it is not something unexpected

 2 ALERT type 1 CIF construction/syntax error, inconsistent or missing data
44 ALERT type 2 Indicator that the structure model may be wrong or deficient
21 ALERT type 3 Indicator that the structure quality may be low
17 ALERT type 4 Improvement, methodology, query or suggestion
 0 ALERT type 5 Informative message, check
```

It is advisable to attempt to resolve as many as possible of the alerts in all categories. Often the minor alerts point to easily fixed oversights, errors and omissions in your CIF or refinement strategy, so attention to these fine details can be worthwhile. In order to resolve some of the more serious problems it may be necessary to carry out additional measurements or structure refinements. However, the purpose of your study may justify the reported deviations and the more serious of these should normally be commented upon in the discussion or experimental section of a paper or in the "special_details" fields of the CIF. checkCIF was carefully designed to identify outliers and unusual parameters, but every test has its limitations and alerts that are not important in a particular case may appear. Conversely, the absence of alerts does not guarantee there are no aspects of the results needing attention. It is up to the individual to critically assess their own results and, if necessary, seek expert advice.

Publication of your CIF in IUCr journals

A basic structural check has been run on your CIF. These basic checks will be run on all CIFs submitted for publication in IUCr journals (*Acta Crystallographica*, *Journal of Applied Crystallography*, *Journal of Synchrotron Radiation*); however, if you intend to submit to *Acta Crystallographica Section C* or *E* or *IUCrData*, you should make sure that full publication checks are run on the final version of your CIF prior to submission.

Publication of your CIF in other journals

Please refer to the *Notes for Authors* of the relevant journal for any special instructions relating to CIF submission.

PLATON version of 02/02/2025; check.def file version of 02/02/2025

Datablock cu_20240720_ys_aucu60_0m - ellipsoid plot

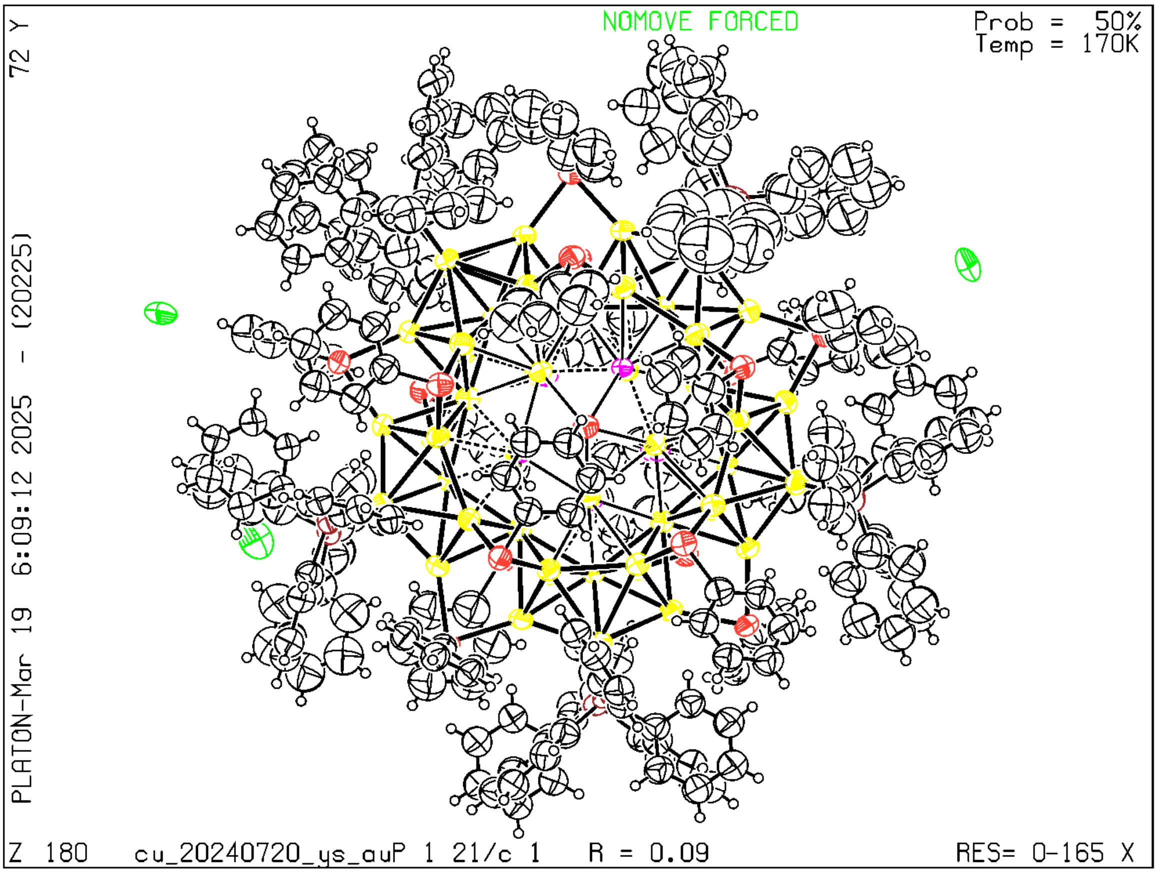

Supplement: Supplementary file 2 — Supporting Information [file ADVS-12-e09283-s002.zip › checkcif for CuxAu61-x.pdf]
